# Would the Real Loneliness Please Stand Up? The Validity of Loneliness Scores and the Reliability of Single-Item Scores

**DOI:** 10.1177/10731911221077227

**Published:** 2022-03-04

**Authors:** Marcus Mund, Marlies Maes, Pia M. Drewke, Antonia Gutzeit, Isabel Jaki, Pamela Qualter

**Affiliations:** 1 University of Klagenfurt, Austria; 2 Friedrich-Schiller-Universität Jena, Germany; 3 KU Leuven, Belgium; 4 Research Foundation Flanders (FWO), Brussels, Belgium; 5 Utrecht University, The Netherlands; 6 The University of Manchester, UK

**Keywords:** loneliness, Rasch-Type Loneliness Scale, University of California Los Angeles Loneliness Scale, single-item measures, validity, nomological net, self-other agreement

## Abstract

Several measures that assess loneliness have been developed for adults. Across three studies, we investigated psychometric features of scores of different versions of the Rasch-Type Loneliness Scale, the University of California Los Angeles Loneliness Scale, and three single-item measures. In Study 1 (*N* = 697 self-ratings, *N* = 282 informant-ratings of 160 targets) and Study 2 (*N* = 1,216 individuals from 608 couples), we investigated convergent validity, self-informant agreement, and nomological nets of the item scores using correlates related to demographic aspects, personality, satisfaction, and network characteristics. In Study 3 (*N* = 411), we estimated a reliability of $r_{xx}>.70$ for scores of three single-item measures of loneliness. Overall, scores of all measures and their nomological nets were highly correlated within and across studies, indicating that the scores of the included measures are all reliable and valid. Recommendations for choosing a loneliness measure are discussed.

Humans are social beings, and they rely and depend on each other (Axelrod & Hamilton, 1981; Baumeister & Leary, 1995). Interpersonal relationships are among the most powerful sources for well-being and health (Baumeister & Leary, 1995; S. Cohen, 2004; Hartup & Stevens, 1997; House et al., 1988). When interpersonal relationships are perceived as deficient regarding quantitative (e.g., network size, number of friends) and/or qualitative aspects (e.g., relationship closeness, trust, intimacy), individuals experience loneliness (Ernst & Cacioppo, 1999; Perlman & Peplau, 1981).

The large body of literature on loneliness that has accumulated is very consistent in demonstrating the negative effects of loneliness on health-related outcomes (for reviews, see Cacioppo et al., 2015; Ernst & Cacioppo, 1999; Hawkley & Cacioppo, 2010; Hawkley & Capitanio, 2015; Heinrich & Gullone, 2006; Holt-Lunstad et al., 2015) and interpersonal outcomes (Cacioppo et al., 2017; Lodder et al., 2017; Mund et al., 2022; Mund & Johnson, 2021; Spithoven et al., 2018). This consistency is to be highlighted given the multitude of loneliness measures that have been developed over the last decades, including unidimensional and multidimensional multi-item scales, various abbreviated versions thereof, and a variety of single-item measures. Although issues of validity have been investigated in prior research, these studies are limited in that they (a) have examined either convergent validity between item scores, but not their correlations with external variables (e.g., Cacioppo, Hawkley, et al., 2006; Grygiel et al., 2013; Iecovich, 2013; Maes et al., 2017; D. Russell et al., 1980), (b) examined the nomological net of scores of only one or two measures at once (Cacioppo, Hawkley, et al., 2006; Shiovitz-Ezra & Ayalon, 2012; Victor et al., 2005), and, when doing so, (c) mostly only considered demographic variables such as age, gender, and education as external correlates of loneliness (Borys & Perlman, 1985; Shiovitz-Ezra & Ayalon, 2012; Victor et al., 2005). Thus, it is unclear whether item scores of different loneliness measures for adults tap into different aspects of loneliness or capture a common core.

In this study, we extend prior work in four ways. First, we simultaneously examined the convergent validity among and the nomological net of scores of the most widely used measures of loneliness in adulthood (Buecker et al., 2020, 2021; Maes et al., 2019; Mund, Freuding, et al., 2020; Pinquart & Sörensen, 2001): the Rasch-Type Loneliness Scale (RTLS) and its facets emotional and social loneliness (de Jong Gierveld & Kamphuis, 1985; de Jong Gierveld & van Tilburg, 2006), the University of California Los Angeles Loneliness Scale (UCLA-LS) in its full (D. Russell et al., 1978, 1980; D. W. Russell, 1996) and two abbreviated versions (Hawkley et al., 2015; Hays & DiMatteo, 1987), and three single-item measures of loneliness. Second, we examined the convergence between scores of self-ratings and informant-ratings of loneliness to extend the assessment of the validity beyond self-reports (McCrae et al., 2004; Podsakoff et al., 2003). Third, in addition to demographic correlates of loneliness, we investigated the nomological net of scores of the various measures using a wide variety of constructs (e.g., Big Five personality traits, domain and life satisfaction, social support, depressiveness, and shyness) to pinpoint similarities and differences between item scores of the selected measures. Fourth, we examined the reliability of scores of three single-item measures to investigate the often-raised criticism that such measures lack reliability (D. Russell, 1982).

To achieve these goals, we conducted three independent studies. Studies 1 and 2 were designed to investigate the validity of item scores and agreement between self-ratings and ratings by close others and to examine the nomological nets of scores of different loneliness measures. In Study 3, we examined the reliability of scores of three single-item measures of loneliness using a procedure developed by Heise (1969) in a study extending over three measurement occasions, each 2 weeks apart.

## Measures of Loneliness

Given its subjective nature (Ernst & Cacioppo, 1999; Perlman & Peplau, 1981), loneliness has most often been assessed using self-reports (Marangoni & Ickes, 1989; D. Russell, 1982). The various self-report measures that have been developed in recent decades primarily differ regarding three aspects (D. Russell, 1982): (a) the dimensionality of the instrument (unidimensional vs. multidimensional), (b) the number of items (i.e., single-item measures vs. multi-item scale), and (c) whether the instrument assesses loneliness in a direct or an indirect way.

### Unidimensional and Multidimensional Measures

Measurement instruments to assess loneliness can be differentiated according to whether they were developed based on the conceptualization of loneliness as a unidimensional construct or as a construct with multiple interrelated facets. Instruments such as the UCLA-LS (D. Russell et al., 1978, 1980; D. W. Russell, 1996) have been designed to assess global loneliness. This notion has been challenged by several studies showing the UCLA-LS scores to be multidimensional and to be accounted for by two (Cramer & Barry, 1999; Knight et al., 1988; Maes et al., 2017; Wilson et al., 1992; Zakahi & Duran, 1982), three (Cacioppo, Hawkley, et al., 2006; Hawkley et al., 2005; McWhirter, 1990; Shevlin et al., 2015), or even five factors (Cacioppo, Hawkley, et al., 2006). By contrast, other studies support the notion of the UCLA-LS scores being essentially unidimensional (Dodeen, 2015; Hartshorne, 1993; Lasgaard, 2007; McDanal et al., 2021; D. Russell et al., 1980; D. W. Russell, 1996). Despite this controversy, we decided to use the UCLA-LS scores as unidimensional in the present work for two main reasons. First, there is no consensual multidimensional solution of UCLA-LS item scores in the literature, and even studies reporting the same number of underlying factors differ markedly in which items are allocated to which factor and how those factors are interpreted. Second, in most research contexts, scores on the UCLA-LS seem to be treated as unidimensional. Specifically, several recent meta-analyses refrained from considering specific aspects of loneliness because there were not enough studies using the UCLA-LS reporting on those facets (Buecker et al., 2020, 2021; Maes et al., 2019; Mund, Freuding, et al., 2020).

Other scales such as the RTLS (de Jong Gierveld & Kamphuis, 1985; de Jong Gierveld & van Tilburg, 2006) have been designed to capture multiple aspects of loneliness such as emotional and social loneliness. Multidimensional approaches offer the opportunity to measure loneliness in a more nuanced fashion and to better understand differential predictors and consequences of those facets. However, when taking the RTLS as an example, surprisingly few studies seem to build on its multidimensionality (for meta-analyses, see Buecker et al., 2020; Mund, Freuding, et al., 2020). This might be due to relatively high correlations between scores of the two facets of the RTLS (Grygiel et al., 2013; Iecovich, 2013; Mund & Neyer, 2016), possibly indicating that the two facets capture actually the same aspects of loneliness. In this study, we will examine both global loneliness as measured by scores of the RTLS and its facets emotional and social loneliness.

### Single Items and Multi-Item Scales

Single-item measures of loneliness have been used frequently (Mund, Freuding, et al., 2020; Pinquart & Sörensen, 2001) and have been attested substantial content validity (D. Russell, 1982). However, their usage has been discouraged for several reasons (Marangoni & Ickes, 1989; D. Russell, 1982). For example, the validity of scores of single-item measures has been questioned due to possible socially desirable responding and lack of standardization (Marangoni & Ickes, 1989; D. Russell, 1982). That is, there is a plethora of single-item measures of loneliness that differ in wording and response format so that it is not fully clear whether scores of all these measures tap into the same construct. Studies comparing scores of single-item with multi-item measures have found a lower prevalence of loneliness when single items are used (Eccles et al., 2020; Victor et al., 2005, but see Shiovitz-Ezra & Ayalon, 2012, for a contrasting finding). Moreover, it seems that the individuals who are classified as “lonely” based on cut-off scores on multi-item scales are not the same individuals who are classified as “lonely” based on a cut-off score applied to single-item measures (Eccles et al., 2020; Shiovitz-Ezra & Ayalon, 2012). Thus, it might be questioned whether scores of single items capture the complexity of the construct in a way scores of multi-item scales do. Apart from prevalence rates, however, the results obtained with scores of single-item measures converge very well with those obtained with scores of multi-item scales regarding demographic aspects (Shiovitz-Ezra & Ayalon, 2012; von Soest et al., 2020), aspects of mean-level and rank-order stability (Mund, Freuding, et al., 2020; Mund, Lüdtke, & Neyer, 2020), personality correlates (Buecker et al., 2020), and health outcomes (Beutel et al., 2017; Eccles et al., 2020), including early mortality (Holt-Lunstad et al., 2015; Shiovitz-Ezra & Ayalon, 2010).

It has also been argued that scores of single items are unreliable and capture more noise than substantial interindividual differences (Marangoni & Ickes, 1989; D. Russell, 1982). In line with this reasoning, a study employing STARTS models (Kenny & Zautra, 2001) found that scores of single-item measures capture more (potentially unreliable) state variance (≈50% state variance) than scores of longer scales such as a six-item version of the RTLS (≈25% state variance; Mund, Lüdtke, & Neyer, 2020). However, it has been shown for scores of single-item measures of subjective well-being that state variance can contain a substantial amount of reliable variance (Lucas & Donnellan, 2012). Similarly, Robins et al. (2001) demonstrated high reliability of scores of a single-item measure of self-esteem ( $r_{xx}=.75$ ) by applying a procedure introduced by Heise (1969). In this approach, the reliability of scores of a single-item measure is estimated based on its pattern of autocorrelation across three measurement points. In this study, we will adopt this approach to estimate the reliability of scores of three single-item measures of loneliness.

### Direct and Indirect Measures of Loneliness

In direct measures of loneliness, the respondents are confronted with the target construct insofar as words such as “lonely” are contained in the questions (e.g., “I am lonely”; “How often have you felt very lonely?”). Indirect measures, by contrast, avoid such references to loneliness (D. Russell, 1982). One item from the UCLA-LS, for example, asks participants to indicate “How often do you feel left out?,” and an item from the RTLS asks participants to rate to what extent they agree with the statement “I experience a general sense of emptiness” (indicative of emotional loneliness).

The reasoning for using indirect measures is to disguise the researchers’ interest in loneliness, thereby avoiding the activation of negative stereotypes and circumventing socially desirable responding (D. Russell, 1982). Previous studies have shown that gender differences in the prevalence of loneliness can be observed when scores of direct measures are used, with men reporting lower levels of loneliness on average than women (Borys & Perlman, 1985). Those gender differences disappear or even reverse when scores of indirect measures are used (Maes et al., 2019). It should be noted that direct measures of loneliness are often single items, which, necessarily, are unidimensional. By contrast, most multi-item measures are indirect measures of loneliness (for an exception, see Rubenstein & Shaver, 1980), and some of them are multidimensional.

## Convergent Validity, Self-Informant Agreement, and Nomological Nets

Previous studies comparing measures of loneliness for adults usually included no more than two measures at once and primarily considered their association with demographic variables (Borys & Perlman, 1985; Cacioppo, Hawkley, et al., 2006; Hughes et al., 2004; Iecovich, 2013; Maes et al., 2017; Shiovitz-Ezra & Ayalon, 2012; Victor et al., 2005; von Soest et al., 2020). Regarding aspects of validity, we extend prior research by (a) considering scores of multiple measures, and their facets, of loneliness for adults, (b) investigating correlations among self-reports and the agreement between self-ratings and informant-ratings on item scores of those measures, and (c) considering a broad range of demographic and psychological variables to investigate similarities and differences in the nomological nets of scores of those measures.

Although several studies have investigated the psychometric properties of scores of multiple loneliness measures for children and adolescents (Cole et al., 2021; Goossens & Beyers, 2002; Maes et al., 2017), studies on scores of loneliness measures for adults have only compared scores of two measures at once (Borys & Perlman, 1985; Cacioppo, Hawkley, et al., 2006; Hughes et al., 2004; Iecovich, 2013; Shiovitz-Ezra & Ayalon, 2012; Victor et al., 2005; von Soest et al., 2020), with a particular focus on the UCLA-LS. However, examining the convergent validity of item scores of multiple measures is important to ensure that scores of all those measures capture interindividual differences in the same construct (Cronbach & Meehl, 1955).

The correlation between self-reports and informant-reports is an important aspect of construct validity (Connelly & Ones, 2010; McCrae et al., 2004; Podsakoff et al., 2003). If scores of an instrument merely capture noise instead of reliable interindividual differences, the correlation with informant-ratings would be low (McCrae, 2015; Podsakoff et al., 2003). Thus, self-informant agreement is indicative of the substance of the measured construct (McCrae et al., 2004). Using a nine-item version of the UCLA-LS, Luhmann et al. (2016) found self-informant agreements for partnered individuals of r=.37,.43, and .66 , when the informants were friends, parents, and partners, respectively. For individuals without a partner, the self-informant agreement with friends amounted to r=.43 and to r=.56 with parents. Using the 20-item UCLA-LS, Lee and Ko (2018) reported a correlation of r=.55 between self-ratings and an aggregated informant-rating of up to three friends in a study with 118 self-(aggregated) informant pairs. Finally, also using the 20-item UCLA-LS, Mearns et al. (2009) reported a correlation of r=.44 between self-ratings and ratings by a friend, roommate, or parent in a study with 74 self-informant pairs. Studies investigating the self-informant agreement in adult samples for scores of other measures than observed using the UCLA-LS are lacking to date.

As another facet of self-informant agreement, it is also possible to examine mean-level differences in self-ratings and informant-ratings of loneliness. While the correlation between self-ratings and informant-ratings pertains to the detectability of interindividual differences, comparing mean levels provides an evaluation of the exact agreement between self-reports and informant-reports (Kim et al., 2019). Previous studies on mean-level differences in loneliness measured using the UCLA-LS have found that parents and friends, but not partners, tend to underestimate the degree of target’s loneliness (Lee & Ko, 2018; Luhmann et al., 2016). No studies have examined mean-level differences between self-ratings and informant-ratings in scores of measures other than the UCLA-LS so far.

The construct validity of the scores of measures investigated in the present work is further examined using a comprehensive nomological net (Cronbach & Meehl, 1955). Previous studies comparing the nomological nets of scores of loneliness measures for adults (Borys & Perlman, 1985; Cacioppo, Hawkley, et al., 2006; Hughes et al., 2004; Iecovich, 2013; Shiovitz-Ezra & Ayalon, 2012; Victor et al., 2005; von Soest et al., 2020) were restricted to a few, mostly demographic aspects. In the present work, we investigated broad nomological nets of scores of the selected measures including demographic aspects, personality characteristics, domain-specific and life satisfaction, and aspects of social networks. Large differences in the nomological nets across item scores would indicate that researchers and practitioners need to choose their measures carefully to make sure that the obtained item scores measure the intended aspects.

## The Present Work

In this article, we present a series of three studies. In Study 1, we investigated the validity (convergence, self-other agreement, and nomological nets) of scores of six measures of loneliness in a sample of young adults. In Study 2, we examined the validity of scores of four partly different measures of loneliness than those used in Study 1. Furthermore, data in Study 2 were collected as part of a larger project on partner relationships; accordingly, informants in Study 2 were romantic partners. In Study 3, we investigated the reliability of scores of three single-item measures of loneliness by designing a short-term prospective study with three measurement occasions each 2 weeks apart. The single studies were not preregistered. Analysis scripts and data used in the analyses are accessible at https://osf.io/7gsfw/.

## Study 1: Validity of Scores of Six Loneliness Measures

### Method

#### Sample

The study was conducted in May 2018 as a cross-sectional online study using the formr survey framework (Arslan et al., 2020). The study was announced on several university mailing lists and on dedicated websites for people interested in psychological studies. The study protocol was approved by the Institutional Review Board of the *Friedrich-Schiller-Universität Jena, Germany (FSV 18/03)*. A total of 697 German-speaking individuals participated in the self-rating part of the study. Among those, 72.17% were female. The majority of the participants were students (73.39%), 15.88% of the participants were employed or self-employed, 2.43% were enrolled in nonacademic education (e.g., vocational learning, traineeship), and 1% were fulfilling a civil service; 7.30% of the participants were unemployed, unable to work because of ill-health, or not working for other reasons (e.g., retired, parental leave). On average, participants were 26.06 years (*SD* = 9.84, *Mdn* = 23) old, ranging from 18 to 99 years. After completion of the survey, participants received personalized feedback on their personality and well-being, and could sign up for a voucher lottery.

Very early in the survey, participants were asked to provide contact details of up to six individuals who could provide ratings of the targets’ personality and well-being. Those contacts were then sent an email and asked to fill in a brief questionnaire. In this way, we obtained 282 informant-ratings on 160 targets. On average, each of the 160 participants was rated by 1.68 informants (*SD* = 1.03). Most of the targets (60.3%) were rated by one, 21.8% were rated by two, 9.9% by three, 6.2% by four, 1.2% by five, and 0.6% by six informants, respectively. On average, the informants were 28.25 years old (*SD* = 13.58, *Mdn* = 22), ranging from 18 to 70 years. Among the informants, 52% were friends of the targets, 23% were kin (e.g., parents, siblings), 18% were the target’s romantic partner, 3% were acquaintances, 3% were colleagues, and 1% indicated to be in another relationship with the target. Given the uneven distribution of types of informants, we aggregated the ratings of all informants in the analyses.

Informants were asked to indicate how well they knew the target using a scale ranging from 0 (*not well*) to 10 (*very well*). On average, informants stated to know the targets very well (8.43, *SD* = 1.54, *Mdn* = 9). Upon completion of the survey, informants were offered the option to sign up for a voucher lottery. Informants were also invited to take part in the self-rating. In this way, it was possible that self-raters also served as informants for other targets. Furthermore, it is possible that some individuals provided informant-ratings for more than one person. The primary data used for the analysis are available at https://osf.io/7gsfw/.

#### Measures

Details on internal consistency, sample items, response formats, descriptive statistics, and zero-order correlations are provided in Supplemental Tables S1 to S3.

#### Loneliness

The loneliness measures were presented in the self-rating and informant-rating parts of the study.

##### Rasch-Type Loneliness Scale

We used the 11-item version of the RTLS (de Jong Gierveld & Kamphuis, 1985) to assess global loneliness and the facets emotional and social loneliness. Internal consistency (coefficient ω ) was high for self-ratings ( $\omega_{Total}=.89,\omega_{Emotional}=.80,\omega_{Social}=.87$ ) and informant-ratings ( $\omega_{Total}=.87,\omega_{Emotional}=.83,\omega_{Social}=.82$ ). The scores of RTLS_*Emotional*_ and RTLS_*Social*_ were highly correlated in self-ratings ( r=.66,p<.001 ) and informant-ratings ( r=.61,p<.001 ).

##### UCLA Loneliness Scale (UCLA-LS_20*Items*_)

We used the German version of the 20-item UCLA-LS (Döring & Bortz, 1993; D. Russell et al., 1980). Internal consistency was high for self-ratings ( ω=.94 ) and informant-ratings ( ω=.93 ).

##### Three-Item Version of the UCLA Loneliness Scale (UCLA-LS_3*Items*_)

We employed a three-item version of the UCLA-LS that has been developed for use in large panel studies (Hawkley et al., 2015; Luhmann & Hawkley, 2016). Internal consistency was satisfactory in self-ratings ( ω=.78 ) and informant-ratings ( ω=.80 ).

##### Direct Single-Item Measure (SI Direct)

As a direct single-item measure of loneliness, we asked the participants to what extent the statement “I feel lonely” applies to them.

##### Indirect Single-Item Measure (SI Indirect)

As an indirect single-item measure of loneliness, we asked participants to indicate their agreement with the statement “I feel alone.”

##### Direct Single-Item Frequency Measure (SI Direct_*frequency*_)

Adapting the SI Direct, we also asked participants about the frequency of loneliness (i.e., “How often do you feel lonely?”).

#### Correlates of Loneliness

All correlates were assessed only in the self-rating part of the study. We examined correlates from the domains of (a) demography, (b) personality, (c) satisfaction, and (d) network characteristics.

##### Demographic Variables

Participants were asked to indicate their age, gender, educational status, and whether they currently have a partner or not.

##### Personality

The *Big Five personality traits* were assessed using a 15-item version of the Big Five Inventory (Hahn et al., 2012). *Self-esteem* was assessed using a three-item version adapted from the Rosenberg Self-Esteem Scale that has been used previously in a large panel study (Huinink et al., 2011). *Depressiveness* was measured using the 10-item State-Trait Depression Scale (Spaderna et al., 2002). The explicit *affiliation motive* was assessed using four items from the Unified Motive Scales (Schönbrodt & Gerstenberg, 2012). We also assessed *need satisfaction* of the affiliation motive by two items from the Basic Need Satisfaction and Frustration Scale (Chen et al., 2015). A product interaction between both variables was calculated to create an index of *frustration of the affiliation motive. Social desirability* was assessed using the 17-item Social Desirability Scale (Stöber, 2001). *Shyness* and *sociability* were assessed using the Shyness and Sociability Scales for adults (Asendorpf & Wilpers, 1998).

#### Satisfaction

Participants were asked to indicate their overall satisfaction with (a) life, (b) education, (c) leisure, (d) friends and social contacts, (e) family, and (f) partner relationship using items taken from larger panel studies (Huinink et al., 2011; Siedler et al., 2008).

#### Network Characteristics

Using single-item measures, participants were asked to indicate the number of (a) close friends, (b) friends on Facebook, (c) persons with whom the participant would be ready to discuss personal or occupational problems (i.e., *emotional support*), (d) persons who could be contacted for practical help (e.g., when relocating; i.e., *instrumental support*), and (e) persons the participant would ask for advice. Furthermore, participants were asked to indicate the frequency of (f) contact with their closest friend(s) and (g) joint activities with their friends.

#### Analytic Procedure

Convergent validity, operationalized as the correlation between scores of the six loneliness measures, was assessed separately for self-ratings and informant-ratings. Furthermore, for scores of each measure, we examined the correlation between self-ratings and informant-ratings. Nomological nets of the item scores were examined by calculating the correlations with the external variables (e.g., demography, personality).

To investigate whether scores of the selected loneliness measures differ in their associations with external variables, we conducted pairwise model comparisons. In the first step of this approach, we entered all item scores and allowed unrestricted correlations between the scores of the loneliness measures and the external variables, resulting in a saturated model. In the next step, we constrained the correlation between scores of two loneliness measures (e.g., RTLS_*Total*_ and UCLA-LS_20*Items*_) and a correlate (e.g., shyness) to be equal. We then compared the fit of this constrained model with the unconstrained model via a χ^2^-difference test with 1 degree of freedom. A significant decrease in model fit in this setup indicated that scores of the two loneliness measures differed significantly in their association with the external variable. Due to the large number of pairwise comparisons, we only consider differences significant when the *p*-value of the χ^2^-difference test was lower than .001. To facilitate the interpretation of the findings, we computed the average absolute correlation ( $\left|\bar{r}\right|$ ) for scores of each loneliness measure and each domain (i.e., demography, personality, satisfaction, network characteristics).

To estimate differences in the correlation patterns between self-ratings and informant-ratings as well as between Study 1 and Study 2, we calculated mean absolute deviations between correlation matrices ( $\left|\bar{∆r}\right|$ ). Furthermore, we compared whether the two correlation matrices (e.g., convergent validity in Study 1 vs. convergent validity in Study 2) are equal to each other. To this end, we used a procedure described by Steiger (1980). Specifically, Steiger (1980) noted that the sum of squared *z*-transformed correlation coefficients follows a χ^2^-distribution under the assumption that the coefficients in the two matrices are equal. For all comparisons between Study 1 and Study 2, we used disattenuated coefficients as input. This was done because the measures in Study 2 were shorter and less internally consistent. As a consequence, the correlations in Study 2 might be smaller than those found in Study 1, although they might follow a very similar pattern. Mean-level differences between self-ratings and informant-ratings were examined using paired-samples *t*-tests and by calculating a standardized mean difference ( d ) to quantify the effect size of the differences.

To examine the similarity of correlation profiles, we calculated the double-entry intraclass correlation ( $ICC_{DE}$ ), which is similar to a one-way random-effects model for exact agreement ( ICC(1) ; McCrae, 2008). Using $ICC_{DE}$ , each element of the compared profiles is entered twice, but in reverse order. For instance, when comparing the correlation between RTLS_*Emotional*_, UCLA-LS_3*Items*_, and SI Direct across Study 1 and Study 2, the correlations observed in Study 1 are first entered as the first number, whereas the coefficients found in Study 2 are entered second. Then, the coefficients observed in Study 2 are entered first and the coefficients observed in Study 1 constitute the second entry. The correlation between those three measures across studies, thus, is based on four entries instead of two. $ICC_{DE}$ is then calculated as the correlation between those four data points. In this way, $ICC_{DE}$ captures differences in both elevation and shape of the compared profiles (McCrae, 2008). In terms of interpretation, $ICC_{DE}$ can range between −1.0 and +1.0, with high positive values indicating a strong similarity between profiles, high negative values indicating a strong dissimilarity between profiles, and an $ICC_{DE}$ around 0 indicating no association between profiles. In all analyses, all variables were treated as manifest variables. All syntax files used in the study are available at https://osf.io/7gsfw/.

### Results and Discussion

#### Preliminary Analyses

Before turning to the key analyses, we conducted some preliminary analyses. First, we examined whether loneliness would be associated with the number of informant-ratings. Across all measures, loneliness was correlated with the number of informant-reports such that individuals with higher scores on loneliness were rated by fewer individuals. On average, this correlation amounted to $\bar{r}=-.23$ , ranging from r=−.26 for the RTLS_*Total*_ to r=−.18 for the SI Direct_*frequency*_. Second, we determined the consensus among the informants regarding the target’s loneliness and examined the reliability of the informant-ratings. We calculated ICC(1) and ICC(1, *k*) to determine the reliability of single informant-ratings and the ratings of all informants together, respectively (Shrout & Fleiss, 1979). ICC(1) amounted to 0.53 for the RTLS_*Total*_, 0.55 for the RTLS_*Emotional*_, 0.19 for the RTLS_*Social*_, 0.53 for the UCLA_20*Items*_, 0.46 for the UCLA_3*Items*_, 0.54 for the SI Direct, 0.47 for the SI Indirect, and 0.40 for the SI Direct_*frequency*_, respectively. ICC(1, *k*) amounted to 0.87 for the RTLS_*Total*_, 0.88 for the RTLS_*Emotional*_, 0.58 for the RTLS_*Social*_, 0.87 for the UCLA_20*Items*_, 0.83 for the UCLA_3*Items*_, 0.88 for the SI Direct, 0.84 for the SI Indirect, and 0.80 for the SI Direct_*frequency*_, respectively. Thus, for all measures except RTLS_*Social*_, the absolute agreement between raters was high and even single informants provided a reliable estimate of the target’s loneliness.

#### Convergent Validity

The intercorrelation between scores of the different measures of loneliness is displayed in Table 1 separately for the self-ratings and aggregated informant-ratings. Overall, the correlations were high for self-ratings ( $\bar{r}=.63$ ) and informant-ratings ( $\bar{r}=.66$ ). Furthermore, the average difference between the two correlation matrices was small ( $|\bar{∆r}|=.08$ ) and the difference between the matrices was not statistically significant ( $\chi^{2}=78.4,df=64,p=.11$ ). Finally, the profile similarity between the coefficients in the two matrices was high ( $ICC_{DE}=.97$ ).

**Table 1.** Study 1: Convergent Validity of Loneliness Scores in Self-Rating and Informant-Rating.

Scale	Rasch-Type Loneliness Scale			UCLA Loneliness Scale		Single items		
	Total	Emotional	Social	20 items	3 items	Direct	Indirect	Direct frequency
RTLS_*Total*_		.95	.89	.85	.76	.67	.68	.43
RTLS_*Emotional*_	.93		.72	.80	.75	.72	.70	.44
RTLS_*Social*_	.89	.66		.77	.65	.49	.53	.34
UCLA-LS_20*Items*_	.87	.77	.82		.70	.58	.64	.41
UCLA-LS_3*Items*_	.73	.75	.57	.73		.61	.65	.39
SI Direct	.61	.66	.43	.57	.59		.74	.48
SI Indirect	.62	.64	.47	.59	.56	.70		.51
SI Direct_*frequency*_	.66	.71	.46	.63	.66	.81	.67	

*Note.* Self-rating (*N =* 657–679) is below the diagonal and informant-rating ( N=160 ) is above the diagonal. All correlations are statistically significant at p<.001 . RTLS = Rasch-Type Loneliness Scale; UCLA-LS = University of Los Angeles California Loneliness Scale; SI = single item.

Within the self-ratings, the correlations between scores of RTLS_*Social*_ and the three single items were lower than other associations and varied between r=.43 and r=.47 . The scores of the UCLA-LS_20*Items*_ were more strongly correlated with the scores of the RTLS_*Total*_ ( r=.87 ) than the UCLA-LS_3*Items*_ ( r=.73 ). Similarly, the scores of the UCLA-LS_20*Items*_ were more strongly correlated with the scores of the RTLS_*Social*_ ( r=.82 ) than the UCLA-LS_3*Items*_ ( r=.57 ). Among the informant-ratings, the scores of the SI Direct_*frequency*_ were least strongly associated with scores of all other measures of loneliness (.34 ≤ r ≤ .51).

Taken together, the findings converge with prior research showing high intercorrelations between scores of different measures of loneliness for adults (Iecovich, 2013; D. Russell et al., 1980). Furthermore, we extended prior research by simultaneously examining the convergent validity of scores of multi-item and single-item instruments, and by demonstrating that the high convergence between item scores also extends to informant-ratings. Thus, we conclude that all the instruments included in Study 1 are appropriate to assess between-person differences in loneliness from an internal (i.e., self-ratings) and an external (i.e., informant-ratings) perspective.

#### Self-Informant Agreement

Table 2 displays the correlations between self-ratings and aggregated informant-ratings. Self-informant agreement on scores of the same scale (shown in the diagonal of Table 2) varied across instruments (.49 ≤ r ≤ .61). The highest self-informant agreement was found for scores of the RTLS_*Total*_ ( r=.61 ) and the UCLA-LS_20*Items*_ ( r=.61 ); the lowest agreement was observed for scores of the SI Direct ( r=.49 ). Across all item scores, the self-informant agreement amounted to $\bar{r}=.51$ . This average correlation converges with other studies in which self-informant agreement in loneliness was investigated (Lee & Ko, 2018; Luhmann et al., 2016; Mearns et al., 2009). Paired-samples *t*-tests indicated no statistically significant differences between self-ratings and informant-ratings obtained with either measurement instrument (.32 ≤ p ≤ .89) and only minor effect sizes of differences between self-ratings and informant-ratings ranging between −0.04 for scores of the RTLS_*Social*_ and 0.07 for scores of the RTLS_*Emotional*_. The only exception were scores of the UCLA-LS_3*Items*_, for which self-ratings were significantly higher than the informant-ratings ( d=0.27,p<.001 ).

**Table 2:** Study 1: Self-Informant Agreement of Scores of Different Loneliness Measures.

	Rasch-Type Loneliness Scale			UCLA Loneliness Scale		Single items		
Scale	I-total	I-emotional	I-social	I-20 items	I-3 items	I-direct	I-indirect	I-direct frequency
S-RTLS_*Total*_	.61							
S-RTLS_*Emotional*_	.56	.57						
S-RTLS_*Social*_	.58	.56	.52					
S-UCLA-LS_20*Items*_	.59	.57	.51	.61				
S-UCLA-LS_3*Items*_	.48	.47	.41	.44	.57			
S-SI Direct	.46	.47	.36	.39	.43	.49		
S-SI Indirect	.51	.52	.39	.42	.50	.51	.55	
S-SI Direct_*Frequency*_	.43	.44	.34	.41	.39	.48	.51	.53

*Note. N* = 159–160 pairs between self-ratings and aggregated informant-ratings. All correlations are statistically significant at p<.001 . I = informant-rating; S = self-rating; RTLS = Rasch-Type Loneliness Scale; UCLA-LS = University of Los Angeles California Loneliness Scale; SI = single item.

To our knowledge, this is the first study that investigated the self-informant agreement on scores of multiple measures of loneliness. The results show that the overall convergence between self-ratings and informant-ratings is high within and across measures. In fact, the amount of self-informant agreement found in the present and previous studies (Lee & Ko, 2018; Luhmann et al., 2016; Mearns et al., 2009) is similar to self-informant agreement in broader personality characteristics such as the Big Five traits (for a meta-analysis, see Connelly & Ones, 2010). Furthermore, the mean differences between self-ratings and informant-ratings are, overall, negligible, indicating that the aggregated informant-ratings provide a relatively accurate description of the target’s loneliness (for similar findings regarding broader personality characteristics, see Hofstee, 1994; Kim et al., 2019; Kolar et al., 1996).

#### The Nomological Nets of Loneliness Scores

Table 3 displays the correlations between scores of the different loneliness measures and a wide variety of correlates pertaining to demography, personality, satisfaction, and aspects of social networks. All correlations were compared pairwise via a model comparison with 1 degree of freedom. We will not go into detail on these model comparisons but instead highlight the overall pattern of results. To facilitate the inter-pretation, we computed scale-wise average absolute correlations ( $\bar{r}$ ) for each block of correlates (i.e., demography, personality, satisfaction, network characteristics). Table 4 shows scale-wise profile correlations derived from the correlations in Table 3.

**Table 3:** Study 1: Nomological Net of Scores of Different Measures of Loneliness (Self-Reports).

Variable	Rasch-Type Loneliness Scale^a^			UCLA Loneliness Scale		Single items		
	Total	Emotional	Social	20 items	3 items	Direct	Indirect	Direct frequency
Demography								
Age	.*05_a_*	*−.03_*b,c*_*	.*13_*d*_*	.*08_*a,d*_*	*−.05_*b,c*_*	*−.09_*b,c*_*	.*00_*a,c,d*_*	*−.10_*b*_*
Gender^b^	−.10_*a,c*_	*−.04_*b*_*	*−.15_*b,c*_*	*−.15_*c*_*	*−.03_*a,b*_*	*−.04_*a,b,c*_*	*−.10_*a,b,c*_*	*−.03_*a,b*_*
Education	−.12*a*	−.10_*b*_	*−.11_*b*_*	*−.11_*a,b*_*	*−.06_*a,b*_*	*−.11_*a,b*_*	*−.10_*a,b*_*	*−.11_*a,b*_*
Partnered	−.19*a,c*	−.21_*b*_	−.13_*b,c*_	−.18_*a,b,c*_	*−.10_*c*_*	*−.25_*a,b*_*	*−.24_*a,b*_*	*−.23_*a,b*_*
Average Absolute Correlation^c^	.11	.10	.13	.13	.06	.12	.11	.12
Personality								
Neuroticism	.29a,c	.34_*b*_	.18_*c*_	.24_*c*_	.37_*a,b*_	.21_*c*_	.19_*c*_	.26_*b,c*_
Extraversion	−.34_*a,d*_	−.30_*c,e*_	−.33_*c,e*_	−.46_*b*_	−.33_*a,e*_	−.19_*d,f*_	−.20_*c,f*_	−.20_*c,f*_
Openness	−.15_*a,d*_	*−.08_*b*_*	*−.20_*c,d*_*	*−.20_*d*_*	*−.11_*a,b,c,d*_*	*−.07_*a,b,c*_*	*−.07_*b,c*_*	*−.03_*b*_*
Agreeableness	−.23_*a,e*_	−.17_*c,d*_	−.27_*d*_	−.29_*a,d*_	−.16_*c,d,e*_	*−.10_*b,c*_*	*−.13_*c,e*_*	*−.08_*b,c*_*
Conscientiousness	−.21_*a*_	−.18_*b*_	−.21_*b*_	−.22_*a,b*_	−.17_*a,b*_	−.18_*a,b*_	−.15_*a,b*_	−.16_*a,b*_
Shyness	.34_*a,d*_	.33_*b,c,e*_	.28_*c,f*_	.39_*a,b*_	.39_*a,b,c*_	.25_*d,e,f*_	.24_*d,f*_	.27_*d,f*_
Sociability	−.30_*a*_	−.19_*c*_	−.37_*d*_	−.42_*d*_	−.20_*c*_	*−.06_*b*_*	*−.13_*b,c*_*	*−.06_*b*_*
Self-Esteem	−.53_*a*_	−.53_*b*_	−.42_*c*_	−.49_*a,b*_	−.53_*a,b,c*_	−.47_*a,b,c*_	−.46_*a,c*_	−.50_*a,b,c*_
Affiliation Motive	−.25_*a*_	−.11_*c*_	−.36_*d*_	−.40_*b,d*_	*−.09_*c,e*_*	*−.04_*c,e*_*	*−.11_*c*_*	*−.00_*e*_*
Unsatisfied Needs	.57_*a*_	.49_*c,d*_	.48_*d,e*_	.57_*a,c*_	.55_*a,c,d*_	.38_*b,e*_	.33_*b*_	.37_*b,e*_
Frustrated Affiliation Motive	−.06_*a*_	*−.01_*b*_*	*−.10_*b*_*	*−.10_*a,b*_*	*−.01_*a,b*_*	*−.06_*a,b*_*	*−.07_*a,b*_*	.*00_*a,b*_*
Depressiveness	.62_*a*_	.61_*c,d*_	.52_*b,d*_	.64_*a,c*_	.58_*a,b,c,d*_	.49_*b*_	.48_*b,e*_	.56_*a,d,e*_
Social Desirability	−.05_*a*_	*−.05_*b*_*	*−.05_*b*_*	*−.04_*a,b*_*	*−.05_*a,b*_*	*−.03_*a,b*_*	*−.01_*a,b*_*	*−.02_*a,b*_*
Average Absolute Correlation^c^	.32	.27	.30	.36	.29	.20	.20	.20
Satisfaction With Domains of Life								
Life	−.53a	−.51_*b*_	−.45_*b*_	−.53_*a,b*_	−.43_*a,b*_	−.49_*a,b*_	−.53_*a,b*_	−.49_*a,b*_
Education	−.36_*a*_	−.34_*b*_	−.32_*b*_	−.36_*a,b*_	−.33_*a,b*_	−.32_*a,b*_	−.30_*a,b*_	−.33_*a,b*_
Leisure	−.45_*a*_	−.41_*b*_	−.40_*b*_	−.48_*a,b*_	−.38_*a,b*_	−.38_*a,b*_	−.40_*a,b*_	−.35_*a,b*_
Friends	−.66_*a*_	−.64_*c*_	−.55_*c,d*_	−.64_*a,c*_	−.60_*a,c,e*_	−.47_*b,c,d*_	−.47_*b,d,e*_	−.43_*b,d*_
Family	−.42_*a*_	−.37_*b*_	−.41_*b*_	−.44_*a,b*_	−.37_*a,b*_	−.39_*a,b*_	−.35_*a,b*_	−.35_*a,b*_
Partner Relationship	−.34_*a*_	−.36_*c*_	−.26_*c,d*_	−.32_*a,c,d*_	−.21_*b,d,e*_	−.33_*a,c,e*_	−.34_*a,c,e*_	−.30_*a,c,e*_
Average Absolute Correlation^c^	.47	.45	.40	.47	.39	.40	.40	.38
Network Characteristics								
Number of Friends (Providing). . .								
Overall	−.32_*a,d*_	−.25_*b,c,e*_	−.35_*c,e*_	−.36_*a,b,c*_	−.24_*b,c,d*_	−.16_*a,d,e*_	−.17_*a,d,e*_	−.16_*d,e*_
On Facebook	*−.04_*a*_*	*−.01_*b*_*	*−.07_*b*_*	*−.08_*a,b*_*	*−.03_*a,b*_*	*−.05_*a,b*_*	*−.05_*a,b*_*	*−.03_*a,b*_*
Help With Problems	−.38_*a,d*_	−.29_*b*_	−.42_*c*_	−.37_*a,c*_	−.23_*b,d*_	−.19_*a,b,d*_	−.20_*a,b,d*_	−.17_*d*_
Instrumental Support	−.37_*a*_	−.31_*b,c*_	−.36_*b*_	−.35_*a,b*_	−.27_*a,b*_	−.22_*a,c*_	−.26_*a,b*_	−.21_*a,c*_
Advice	−.22_*a*_	−.18_*b*_	−.23_*b*_	−.24_*a,b*_	−.16_*a,b*_	−.17_*a,b*_	−.19_*a,b*_	−.19_*a,b*_
Frequency of								
Contact	−.22_*a,e*_	−.15_*b,d*_	−.27_*b,c*_	−.27_*a,c*_	−.13_*d,e*_	*−.02_*e,f*_*	*−.08_*a,d,f*_*	*−.02_*b,e,f*_*
Joint Activities	−.41_*a,d*_	−.34_*c*_	−.42_*c*_	−.45_*a*_	−.31_*b,c,d*_	−.17_*b,d*_	−.15_*b*_	−.12_*b*_
Average Absolute Correlation^b^	.28	.22	.31	.31	.20	.14	.16	.13

*Note.* Different subscripts indicate a significantly different ( p<.001 ) correlation as judged by the results of a χ^2^-difference test with 1 degree of freedom. Italicized correlations have a *p*-value ≥. 01. RTLS = Rasch-Type Loneliness Scale.

a Due to the high correlation between the total score on the RTLS and its subfacets emotional and social loneliness, we ran different batches of models. In one batch, we compared the correlations between scores of the full RTLS and scores of all other scales; in the second batch, we compared the correlations between scores of the emotional and social loneliness facets with scores of all other scales. Hence, we could not test whether correlations differ between the full RTLS and its subfacets. ^b^Gender was coded as 1 = female and 0 = male/other. ^c^Average correlations were calculated based on the absolute *z*-transformed correlations. After averaging, *z*-scores were retransformed to r .

**Table 4:** Study 1: Profile Correlations for the Association Between Loneliness Scores and Correlates.

Scale	Rasch-Type Loneliness Scale			UCLA Loneliness Scale		Single items		
	Total	Emotional	Social	20 items	3 items	Direct	Indirect	Direct frequency
RTLS_*Total*_								
RTLS_Emotional_	.98							
RTLS_*Social*_	.97	.93						
UCLA-LS_20*Items*_	.98	.94	.97					
UCLA-LS_3*Items*_	.96	.98	.92	.93				
SI Direct	.88	.94	.84	.82	.93			
SI Indirect	.89	.94	.87	.84	.92	.99		
SI Direct_*frequency*_	.87	.94	.82	.80	.93	.99	.98	

*Note.* The table shows the correlations of the profiles of different loneliness scores with regard to the correlates displayed in Table 3. Profile correlations are based on *z*-scores and were calculated using the double-entry ICC. RTLS = Rasch-Type Loneliness Scale; UCLA-LS = University of Los Angeles California Loneliness Scale; SI = single item; ICC = intraclass correlation.

#### Demography

Item scores of all measures were only modestly related to demographic aspects. Average absolute correlations ranged from $|\bar{r}|=.06$ for scores of the UCLA-LS_3*Items*_ to $|\bar{r}|=.13$ for scores of the UCLA-LS_20*Items*_ and the RTLS_*Social*_.

#### Personality

The average absolute correlations ranged from $|\bar{r}|=.20$ for scores of the three single-item measures to $|\bar{r}|=.36$ for scores of the UCLA-LS_20*Items*_. These differences held also when computing $\left|\bar{r}\right|$ for disattenuated correlations (RTLS_*Total*_ = .41, RTLS_*Emotional*_ = .39, RTLS_*Social*_ = .39, UCLA-LS_20*Items*_=.45, UCLA-LS_3*Items*_ = .41, SI Direct = .29, SI Indirect = .27, and SI Direct_*frequency*_ = .29—for the single items, we used the reliability estimated in Study 3 as correction), accounting for the possibility that differences in $\left|\bar{r}\right|$ occurred due to differences in the reliability of the item scores rather than the true association with the correlates. These differences were probably due to substantially lower correlations between scores of the single items and personality aspects such as openness, agreeableness, conscientiousness, sociability, and need frustration. Furthermore, the correlations suggested that scores of the UCLA-LS_20*Items*_ were more strongly related to aspects associated with extraversion (i.e., sociability, shyness, affiliation motive) than scores of other instruments.

#### Satisfaction With Domains of Life

The average absolute correlations between loneliness scores and domains of satisfaction ranged from $|\bar{r}|=.38$ for scores of the SI Direct_*frequency*_ to $|\bar{r}|=.47$ for scores of the RTLS_*Total*_ and the UCLA-LS_20*Items*_. Scores of the RTLS_*Total*_, RTLS_*Emotional*_, and UCLA-LS_20*Items*_ stood out in this regard with average absolute correlations between $|\bar{r}|=.45$ and $|\bar{r}|=.47$ , whereas all other item scores varied on a somewhat lower level with .38 ≤ $\left|\bar{r}\right|$ ≤ .40 The largest differences between item scores emerged for the association with satisfaction with friends and social contacts. Scores of single-item measures were less strongly associated with this aspect (.43 ≤ $\left|\bar{r}\right|$ ≤ .47) than scores of the multi-item instruments (.55 ≤ $\left|\bar{r}\right|$ ≤ .66).

#### Network Characteristics

Across all item scores, loneliness was associated with smaller friendship and support networks. All scores were uncorrelated with the self-reported number of friends on Facebook. The average absolute correlations ranged from $|\bar{r}|=.13$ for scores of the SI Direct_*frequency*_ to $|\bar{r}|=.31$ for scores of the RTLS_*Social*_ and the UCLA-LS_20*Items*_. Thus, as with the personality correlates, scores of the UCLA-LS_20*Items*_ seem to tap into aspects related to sociability and support. The average absolute correlations with network characteristics were particularly low for the single items ( $|\bar{r}|=.13,.14,.16$ for scores of the SI Direct_*frequency*_, SI Direct, and SI Indirect, respectively) and were somewhat higher for scores of the UCLA-LS_3*Items*_ ( $|\bar{r}|=.20$ ) and the RTLS_*Emotional*_ ( $|\bar{r}|=.22$ ). Except for the number of friends providing instrumental support and advice, scores of the single items were markedly less strongly correlated with network characteristics than scores of the other instruments; these differences were particularly strong with regard to the frequency of contact with friends. In this case, scores of the single items were not significantly correlated with this variable, whereas scores of the multi-item scales were.

#### Profile Similarity

The correlation between the profiles of the loneliness scores and the correlates examined in Study 1 are displayed in Table 4. On average, the profiles of the item scores were very similar to each other, indicating that their nomological nets overlap to a large extent. Nevertheless, it might be noted that the profile correlation between scores of the single items and scores of the RTLS_*Total*_ (.87 ≤ ICCDE ≤ .89), RTLS_*Social*_ (.82 ≤ ICCDE ≤ .87) and UCLA-LS_20*Items*_ (.80 ≤ ICCDE ≤ .84) was relatively low. By contrast, the profile correlations were substantially higher with the RTLS_*Emotional*_ ( $ICC_{DE}=.94$ ) and the UCLA-LS_3*Items*_ (.92 ≤ ICCDE ≤ .93). Similarly, the profile of the UCLA-LS_3*Items*_ was less strongly correlated with the profile of the RTLS_*Social*_ ( $ICC_{DE}=.92$ ) and the UCLA-LS_20*Items*_ ( $ICC_{DE}=.93$ ) than with the RTLS_*Emotional*_ ( $ICC_{DE}=.98$ ). By contrast, and resembling the findings presented in Table 3, the profile of the UCLA-LS_20*Items*_ was closely related to the RTLS_*Social*_ ( $ICC_{DE}=.97$ ).

#### Summary and Conclusion

Overall, the results of Study 1 show that (a) all scores of measures of loneliness are highly correlated in the self-reports and informant-reports, (b) self-informant agreement was high for scores of all measures, and (c) scores of all measures have a similar nomological net. These findings suggest that scores of all measures tap into the same underlying latent construct and that there are no strong qualitative differences between the measures. It should be noted, though, that the nomological net of the scores obtained using single-item measures and—to a lesser extent—scores of the UCLA-LS_3*Items*_ overlapped less strongly with related phenomena pertaining to personality (e.g., extraversion, sociability) and social networks (e.g., contact frequency). It could be argued that scores of these measures provide purer assessments of loneliness. However, it could also be argued that these lower correlations are due to the lower reliability of scores of these measures (Marangoni & Ickes, 1989; D. Russell, 1982). Indeed, the UCLA-LS_3*Items*_ had a lower internal consistency in the present study ( ω=.78 ) than the UCLA-LS_20*Items*_ ( ω=.94 ) and the RTLS_*Total*_ ( ω=.89 ).

## Study 2: Validity of Scores of Four Loneliness Measures

### Method

#### Sample

The data used in Study 2 were taken from a larger online study on personality and partner relationships (*1 citation removed for masked review*). The study protocol was approved by the Institutional Review Board of the *Friedrich Schiller University Jena (FSV 18/47)*. The larger study combines features of longitudinal and diary methods in that couples are asked every 3 months about aspects of their own and their partner’s personality, evaluations of the partner relationship, and aspects related to partner perception and communication patterns. In addition, participants are invited in February and March each year to participate in a diary period extending over 28 days in total, separated by a 3-week break after the first 14 days. Participants were recruited via advertisements on social media platforms, several university mailing lists, and online forums visited by people interested in research participation (Mund & Drewke, 2020).

In this study, we use data from the first five longitudinal waves. These waves were conducted in January 2020, April 2020, July 2020, October 2020, and January 2021, respectively. We included data of couples when they participated for the first time. That is, for the purpose of this study, we did not build on the longitudinal nature of the study, but on its dyadic nature and the large sample size. Accordingly, when a couple participated more than once, we only used data from the first time both partners provided data. This first-time participation could have been anytime during the five waves. In this way, we included data from 1,216 individuals (608 mixed-sex couples). Of those 608 couples, 136 entered the study in January 2020, 48 new couples entered in April 2020, 141 new couples entered in July 2020, 219 new couples entered in October 2020, and 64 new couples entered in January 2021.

Despite couples entering the study at different measurement occasions, analyses of variance indicated no mean-level differences across waves for scores of the UCLA-LS_8*Items*_, F(4,1208)=1.07,p=.37 , UCLA-LS_3*Items*_, F(4,1207)=1.29,p=.27 , or the SI Direct, F(3,753) = 1.87, p = .13. There was a difference, however, for the RTLS_*Total*_ ( F(4,1193)=3.61,p=.006 . Post hoc Tukey tests revealed that the mean of the RTLS_*Total*_ was significantly higher in October 2020 compared with April 2020 (∆ = 0.26, p = .013). All other pairwise comparisons, however, were not statistically significant (.075 ≤ p ≤ .999). This difference in the RTLS_*Total*_ is attributable to differences between waves in the RTLS_*Social*_ ( F(4,1193)=3.37,p=.009 ). Tukey post hoc tests indicated that social loneliness was higher in October 2020 than in January 2020 (∆ = 0.18, p = .036) and April 2020 (∆ = 0.27, p = .030). All other pairwise comparisons were not statistically significant (.299 ≤ p ≤ .998). No difference between waves was observed for RTLS_*Emotional*_, F(4,1193)=2.19,p=.068 .

The same pattern of results was observed when comparing data collected before the COVID-19 pandemic (i.e., Wave 1, January 2020, *N* = 272) with data collected during the pandemic (i.e., from Wave 2 conducted in April 2020 onward; *N* = 944). By using independent-samples Welch tests, we found no differences in the levels of loneliness before and during the pandemic for the UCLA-LS_20*Items*_, $t(462.67)=-1.84,p=.068,{\mathrm{Hedges}}^{\prime }s\;g=-0.13$ , the UCLA-LS_3*Items*_, $t(451.77)=-0.36,p=.722,{\mathrm{Hedges}}^{\prime }s\;g=-0.01$ , or the SI Direct, *t*(560.37) = −0.61, *p* = .541, Hedges"s *g* = −0.04. For the RTLS_*Total*_, however, there was a stati-stically significant difference in the item scores, *t*(429.27) = −2.00, *p* = .046, Hedges’s *g* = −0.14. As before, this difference can be attributed to scores of the RTLS_*Social*_ being higher during the pandemic, *t*(420.87) = –2.18, $p=.030,{\mathrm{Hedges}}^{\prime }s\;g=-0.15$ , whereas no difference emerged for RTLS_*Emotional*_, *t*(426.03) = –1.24, *p* = .216, ${\mathrm{Hedges}}^{\prime }s\;g=-0.08$ . In sum, the few observed differences were small in size (J. Cohen, 1992; Gignac & Szodorai, 2016) and inconsistent across loneliness measures. Thus, we conclude that there are no strong differences in the mean levels of loneliness before and during the pandemic in the present sample (for similar findings, see Bu et al., 2020; Luchetti et al., 2020; Ray, 2021; for a review, see Buecker & Horstmann, 2021). Accordingly, we decided to proceed with the analyses using the pooled data set.

On average, participants were 26.69 years old ( SD=4.86 ) and had been engaged in their current relationship for 40.85 months on average ( SD=40.91,Mdn=28 ), with a range from 2 weeks to 44 years. The majority of couples lived in a noninstitutionalized relationship (81.25%), and 11.35% were married. The remaining participants indicated other relationship forms (e.g., in registered partnership) or preferred not to answer the question. A minority of participants (5.83%) reported to have children. Of those, 81.25% reported to have one child, 11.35% reported to have two children, and 7.40% reported to have three or more children. As highest educational degree, 0.08% of participants reported to have finished primary school, 0.99% reported to have achieved a secondary school diploma, 6.71% have achieved a high school diploma, 6.13% have achieved an entrance qualification for a University of Applied Sciences (Fachabitur), 36.87% have earned a University entrance qualification (Abitur), 47.97% have earned a diploma from a University or a University of Applied Sciences, and 1.24% have completed a PhD. The remaining 0.01% of participants indicated “other” degrees or preferred not to answer this question. Almost half of the participants (48%) were students at the time of study participation. A subset of the data without any personal information (age, gender, etc.) is available from https://osf.io/7gsfw/.

#### Measures

Details on internal consistency, sample items, response formats, descriptive statistics, and zero-order correlations are provided in Supplemental Tables S4 to S6. Differences between samples of Study 1 and Study 2 regarding their average levels of loneliness are discussed in Section 3 of the Supplement, including Supplemental Tables S7 and S8.

#### Loneliness

For all loneliness measures, we obtained a self-rating and a partner-rating. That is, each participant rated and was rated by their partner on each loneliness measure.

##### Rasch-Type Loneliness Scale

In Study 2, we used the six-item version of the RTLS (de Jong Gierveld & van Tilburg, 2006), which also allows to differentiate between emotional and social loneliness. The correlation between scores of the two facets was less strong than in Study 1 for both the self-rating ( r=.46,p<.001 ) and the partner-rating ( r=.47,p<.001 ). Internal consistency was high for scores of the full scale and its facets in the self-rating ( $\omega_{Total}=.78,\omega_{Emotional}=.68,\omega_{Social}=.83$ ) as well as in the partner-rating ( $\omega_{Total}=.79,\omega_{Emotional}=.66,\omega_{Social}=.85$ ).

##### UCLA Loneliness Scale (UCLA-LS_8*Items*_)

We used the German version of the eight-item UCLA-LS (Hays & DiMatteo, 1987). Internal consistency was high for self-rating ( ω=.80 ) and partner-rating ( ω=.81 ).

##### Three-Item Version of the UCLA Loneliness Scale (UCLA-LS_3*Items*_)

As in Study 1, we employed a three-item version of the UCLA-LS as proposed by Hawkley et al. (2015). Internal consistency was satisfactory in the self-rating ( ω=.77 ) and the informant-rating ( ω=.81 ).

##### Direct Single-Item Measure (SI Direct)

As a direct single-item measure of loneliness, we used the same measure as employed in Study 1. Self-ratings of this item were collected in January 2020, April 2020, July 2020, and January 2021, but not in October 2020. Partner-ratings were collected at all measurement occasions.

#### Correlates of Loneliness

As in Study 1, we used variables from several domains to examine the nomological net of scores of the loneliness measures. Specifically, we used correlates from the domains of (a) demography, (b) personality, and (c) satisfaction.

##### Demographic Variables

Participants were asked to indicate their age, gender, educational status, and their relationship duration.

##### Personality

The *Big Five* were assessed using a 15-item version of the Big Five Inventory Version 2 (Rammstedt et al., 2020; Soto & John, 2017). *Self-esteem* was assessed using the 10-item Rosenberg Self-Esteem Scale (von Collani & Herzberg, 2003). *Depressiveness* was measured using the five negatively worded items from the State-Trait Depression Scale (Spaderna et al., 2002). The explicit *affiliation motive* was assessed using two items from the Unified Motive Scales (Schönbrodt & Gerstenberg, 2012). *Shyness* and *sociability* were assessed using the Shyness and Sociability Scales for adults (Asendorpf & Wilpers, 1998). Sociability was only assessed at the first three measurement waves (January, April, and July 2020, respectively).

#### Satisfaction

Participants were asked to indicate their overall satisfaction with (a) life, (b) education, (c) leisure, (d) friends and social contacts, and (e) family using single items (Huinink et al., 2011; Siedler et al., 2008). *Relationship satisfaction* was assessed with the seven-item Relationship Assessment Scale (Hendrick, 1988; Sander & Böcker, 1993).

### Results and Discussion

#### Convergent Validity

The intercorrelation between scores of the loneliness measures used in Study 2 is displayed in Table 5 separately for self-ratings (below the diagonal) and partner-ratings (above the diagonal). For self-ratings, intercorrelations were high ( $\bar{r}=.59$ ) and comparable to Study 1 ( $\bar{r}=.63$ ). The average correlation for the partner-ratings was lower ( $\bar{r}=.47$ ) than the average correlation in the self-ratings of Study 2 and the informant-ratings of Study 1 ( $\bar{r}=.66$ ). This relatively lower convergence seems to be due to the partner-rated SI Direct. For this item, the correlation with other measures was weak, ranging from r=.07 with partner-rated RTLS_*Social*_ to r=.15 with partner-ratings on the UCLA-LS_8*Items*_. Without the SI Direct, the average correlation in the partner-ratings increased to $\bar{r}=.60$ —a value which is comparable to self-ratings in Study 2 and informant-ratings in Study 1.

**Table 5.** Study 2: Convergent Validity Loneliness Scores in Self- and Informant-Rating.

Scale	Rasch-Type Loneliness Scale			UCLA Loneliness Scale		SI direct
	Total	Emotional	Social	8 items	3 items	
RTLS_*Total*_		.85	.86	.74	.67	.12
RTLS_*Emotional*_	.86		.47	.61	.69	.13
RTLS_*Social*_	.84	.46		.65	.45	.*07*
UCLA-LS_8*Items*_	.71	.63	.58		.74	.15
UCLA-LS_3*Items*_	.65	.69	.42	.76		.12
SI Direct	.66	.66	.45	.67	.66	

*Note.* Self-rating ( N=757−1,212 ) is below the diagonal, and informant-rating ( N=1,181−1,196 ) is above the diagonal. Italicized correlations have a *p*-value ≥.001 . SI = single item; RTLS = Rasch-Type Loneliness Scale; UCLA-LS = University of Los Angeles California Loneliness Scale.

The average difference between the self-ratings and partner-ratings in Study 2 was $\left|\bar{∆r}\right|$ = .19 . Without the SI Direct, the two correlation matrices were nearly identical ( $\left|\bar{∆r}\right|$ =.03). The same pattern emerges when testing whether the two correlation matrices are equal. Including the SI Direct, the two matrices were significantly different ( $\chi^{2}=2412.95,df=36,p<.001$ ). By contrast, there was no statistically significant difference without SI Direct ( $\chi^{2}=31.96,df=25,p=.16$ ). The profile correlation between self-ratings and partner-ratings amounted to $ICC_{DE}=.67$ for scores of all measures and increased to $ICC_{DE}=.99$ when SI Direct was left out.

Finally, we compared the average differences and profile correlations between Study 1 and Study 2 separately for the self-ratings and informant-ratings for the measures included in both studies (i.e., ${\mathrm{RTLS}}_{11Items/6Items}$ and facets, ${\mathrm{UCLA-LS}}_{20Items/8Items}$ , ${\mathrm{UCLA-LS}}_{3Items}$ , SI Direct). The absolute difference in the self-ratings between Study 1 and Study 2 amounted to $\left|\bar{∆r}\right|$ = .16. Both with ( $\chi^{2}=1809.22,df=36,p<.001$ ) and without ( $\chi^{2}=1114.47,df=25,p<.001$ ) SI Direct, the difference in the correlation matrices was statistically significant. For the informant-ratings, $\left|\bar{∆r}\right|$ amounted to .31 when SI Direct was included and to .22 when SI Direct was left out. The difference in the correlation matrices was again statistically significant with ( $\chi^{2}=649.35,df=36,p<.001$ ) and without ( $\chi^{2}=328.59,df=25,p<.001$ ) SI Direct. Regarding the profile correlations between Study 1 and Study 2, $ICC_{DE}$ amounted to .86 for the self-ratings, indicating a very high similarity of the profiles across studies despite the fact that we used different measures in Study 2. For the informant-ratings, the profile similarity amounted to $ICC_{DE}=.65$ and was even higher when the SI Direct was excluded ( $ICC_{DE}=.83$ ).

Taken together, the results regarding the convergent validity of scores of different loneliness measures obtained in Study 2 converged very well with the results observed in Study 1. Across the two studies, the correlation profiles were very similar and the differences in the average correlations were small to moderate. Unlike Study 1, the SI Direct turned out to be somewhat problematic in the partner-rating of Study 2 in the sense that it showed only modest correlations with scores of other partner-rated loneliness measures. One possible explanation for this finding is that the SI Direct forced individuals to explicitly rate their partners as “lonely,” which might threaten the self-concept of the rating partner. In Study 1, such effects might have been removed through the aggregation of the informant-ratings across multiple raters.

#### Self-Informant Agreement

The agreement between self-ratings and partner-ratings are displayed in Table 6. The diagonal shows the agreement on the same instrument, ranging between r=.22 for scores of the SI Direct and r=.55 for scores of the ${\mathrm{UCLA-LS}}_{8Items}$ . Apart from the SI Direct, however, the correlations in the diagonal are close to each other with .42 ≤ r ≤ .55. With the exception of ${\mathrm{RTLS}}_{Social}$ , the agreement between self-rated SI Direct and partner-rated measures was almost twice as high (.42 ≤ r ≤ .44) as the correlation with partner-rated SI Direct ( r=.22 ). Across all item scores, self-informant agreement amounted to $|\bar{r}|=.39$ with and to $|\bar{r}|=.43$ without the SI Direct, respectively. These coefficients were lower than the average correlation observed in Study 1 ( $|\bar{r}|=.51$ ). It should be noted, though, that we employed shorter measures of the UCLA-LS and RTLS in Study 2 that evinced a lower internal consistency. When correcting for attenuation, the self-informant agreement for scores of the ${\mathrm{UCLA-LS}}_{8Items}$ , for example, increased to $\hat{r}=.68$ , which is very similar to a corrected self-informant agreement for scores of the ${\mathrm{UCLA-LS}}_{20Items}$ in Study 1 ( $\hat{r}=.65$ ).

**Table 6.** Study 2: Self-Informant Agreement of the Different Loneliness Scores.

	Rasch-Type Loneliness Scale			UCLA Loneliness Scale		I-SI direct
	I-total	I-emotional	I-social	I-8 items	I-3 items	
${\mathrm{S-RTLS}}_{Total}$	.53					
${\mathrm{S-RTLS}}_{Emotional}$	.47	.49				
${\mathrm{S-RTLS}}_{Social}$	.43	.32	.42			
${\mathrm{S-UCLA-LS}}_{8Items}$	.48	.42	.41	.55		
${\mathrm{S-UCLA-LS}}_{3Items}$	.43	.45	.30	.45	.48	
S-SI Direct	.42	.44	.26	.42	.44	.22

*Note. N* = 742–1,196 pairs of self-ratings and partner-ratings. All correlations are statistically significant at p<.001 . I = informant-rating; SI = single item; S = self-rating; RTLS = Rasch-Type Loneliness Scale; UCLA-LS = University of Los Angeles California Loneliness Scale.

In contrast to Study 1, paired-samples *t*-tests indicated consistent statistically significant differences between self-ratings and informant-ratings (all *p*s < .001). These differences were moderate in size, ranging between −0.31 for the ${\mathrm{RTLS}}_{Social}$ and −0.14 for the ${\mathrm{UCLA-LS}}_{3Items}$ . For all measures, the scores of the informant-reports were larger than the self-report scores, indicating that individuals described their partners as lonelier than they described themselves. This finding is in contrast to a previous study reporting no difference between self-rated and partner-rated loneliness using a nine-item version of the UCLA-LS in a sample of N=132 partnered students (Luhmann et al., 2016). A meta-analysis on differences in self-reports and informant-reports of broader personality characteristics has found that closer informants typically see targets in a more favorable light (e.g., targets report higher scores on neuroticism than informants; Kim et al., 2019).

Comparing self-informant agreement between Study 1 and Study 2, we found a weak profile correlation of $ICC_{DE}=.16$ . However, the average difference between the matrices was only small ( $\left|\bar{∆r}\right|$ = .11). The difference between the matrices was statistically significant both with ( $\chi^{2}=95.04,df=36,p<.001$ ) and without ( $\chi^{2}=77.29,df=25,p<.001$ ) SI Direct. Thus, small shifts in the profiles might have led to low profile similarity, whereas the actual coefficients do not differ much between Study 1 and Study 2.

#### The Nomological Nets of Loneliness Measures

The correlations between the different loneliness item scores and external correlates are displayed in Table 7. As in Study 1, we focus on the average absolute correlations ( $\left|\bar{r}\right|$ ) when discussing the results. Table 7 additionally contains the results of pairwise model tests with 1 degree of freedom for each scale and each correlate. The similarity between the correlation profiles of the different loneliness scores with external correlates is displayed in Table 8.

**Table 7.** Study 2: Nomological Nets of Different Measures of Loneliness (Self-Reports).

Variable	Rasch-Type Loneliness Scale^a^			UCLA Loneliness Scale		SI direct
	Total	Emotional	Social	8 items	3 items	
Demography						
Age	*−.07_a_*	*−.15_b_*	.*04_c_*	*−.07_a,b,c_*	*−.15_a,b_*	*−.07_a,b,c_*
Gender^b^	*−.01_a_*	.06_*c*_	*−.09_d_*	.*05_a,c_*	.*17_b_*	.*12_b,c_*
Education	−.13_*a*_	−.10_*b*_	−.11_*b*_	*−.01_b_*	*−.01_b_*	*−.04_a,b_*
Relationship Duration	*−.06_a_*	−.10_*b*_	.*01_b_*	*−.01_a,b_*	*−.06_a,b_*	*−.03_a,b_*
Average Absolute Correlation^c^	.07	.10	.06	.04	.10	.06
Personality						
Neuroticism	.46_*a,d*_	.54_*b*_	.22_*c*_	.41_*a*_	.53_*b,d*_	.49_*a,b*_
Extraversion	−.17_*a*_	*−.09_c_*	*−.20_c,d_*	*−.30_b,d_*	*−.17_a,c_*	*−.15_a,c_*
Openness	.*05_a_*	.*10_b_*	*−.02_b_*	.*04_a,b_*	.*07_a,b_*	.*08_a,b_*
Agreeableness	−.22_*a,f*_	−.14_*c,e*_	−.24_*c,d*_	−.27_*a,d*_	*−.09_b,e_*	*−.16_b,c,f_*
Conscientiousness	−.20_*a*_	−.19_*b*_	−.15_*b*_	−.15_*a,b*_	−.18_*a,b*_	−.15_*a,b*_
Shyness	.23_*a*_	.23_*c*_	.16_*c*_	.36_*b*_	.28_*a,b,c*_	.23_*a,c*_
Sociability	−.14_*a*_	*−.01_c_*	*−.24_b_*	*−.30_b_*	*−.05_a,c_*	*−.08_a,c_*
Self-Esteem	−.54_*a*_	−.55_*b*_	−.36_*c*_	−.48_*a,b*_	−.51_*a,b*_	−.52_*a,b*_
Affiliation Motive	−.12_*a*_	.*05_c_*	*−.26_b_*	*−.24_b_*	.*01_c_*	*−.05_a,c_*
Depressiveness	.56_*a*_	.60_*b*_	.33_*c*_	.55_*a,b*_	.58_*a,b*_	.58_*a,b*_
Average Absolute Correlation^c^	.28	.27	.22	.32	.26	.26
Satisfaction With Domains of Life						
Life	−.39_*a*_	−.35_*b*_	−.30_*b*_	−.34_*a,b*_	−.27_*b*_	−.31_*a,b*_
Education	−.31_*a*_	−.30_*b*_	−.21_*b*_	−.23_*a,b*_	−.23_*a,b*_	−.25_*a,b*_
Leisure	−.32_*a*_	−.29_*b*_	−.25_*b*_	−.33_*a,b*_	−.29_*a,b*_	−.30_*a,b*_
Friends	−.50_*a,c*_	−.39_*b,c*_	−.45_*b*_	−.48_*a,b*_	−.39_*c,d,e*_	−.42_*a,b,e*_
Family	−.29_*a*_	−.25_*b*_	−.23_*b*_	−.23_*a,b*_	−.22_*a,b*_	−.24_*a,b*_
Partner Relationship	−.28_*a*_	−.27_*b*_	−.21_*b*_	−.24_*a,b*_	−.23_*a,b*_	−.29_*a,b*_
Average Absolute Correlation^c^	.35	.31	.28	.31	.27	.30

*Note.* Different subscripts indicate a significantly different ( p<.001 ) correlation as judged by the results of a χ^2^-difference test with 1 degree of freedom. Italicized correlations have a *p*-value ≥.01 . SI = single item; RTLS = Rasch-Type Loneliness Scale.

a Due to the high correlation between scores of the total score on the RTLS and its subfacets emotional and social loneliness, we ran different batches of models. In one batch, we compared the correlations between scores of the full RTLS and all other scales, and in the second batch, we compared the correlations between scores of the emotional and social loneliness facets with all other scales. Hence, we could not test whether correlations differ between scores of the full RTLS and its subfacets. ^b^Gender was coded as 1 = female and 0 = male/other. ^c^Average correlations were calculated based on the absolute *z*-transformed correlations. After averaging, *z*-scores were retransformed to r .

**Table 8:** Study 2: Profile Correlations Between Loneliness Scores and Correlates.

Scale	Rasch-Type Loneliness Scale			UCLA Loneliness Scale		SI direct
	Total	Emotional	Social	8 items	3 items	
${\mathrm{RTLS}}_{Total}$						
${\mathrm{RTLS}}_{Emotional}$	.97					
${\mathrm{RTLS}}_{Social}$	.88	.77				
${\mathrm{UCLA-LS}}_{8Items}$	.96	.91	.90			
${\mathrm{UCLA-LS}}_{3Items}$	.95	.98	.79	.93		
SI Direct	.98	.98	.84	.95	.99	

*Note.* The table shows the correlations of the profiles of different loneliness scales with regard to the correlates displayed in Table 7. Profile correlations are based on *z*-scores and were calculated using the double-entry ICC. SI = single item; RTLS = Rasch-Type Loneliness Scale; UCLA-LS = University of Los Angeles California Loneliness Scale; ICC = intraclass correlation.

#### Demography

The absolute average correlations varied only slightly between the loneliness measures with (.04 ≤ $\left|\bar{r}\right|$ ≤ .10. In many cases, the correlations between the demographic aspects and the loneliness scores were not statistically significant.

#### Personality

Compared to Study 1, the absolute average correlations in Study 2 were substantially lower, ranging from $|\bar{r}|=.22$ for ${\mathrm{RTLS}}_{Social}$ to $|\bar{r}|=.32$ for ${\mathrm{UCLA-LS}}_{8Items}$ . When calculating disattenuated correlations, the $\left|\bar{r}\right|$ values were very close to those observed in Study 1 ( ${\mathrm{RTLS}}_{Total}$ = .39 vs. .41, ${\mathrm{RTLS}}_{Emotional}$ = .39 vs. .39, ${\mathrm{RTLS}}_{Social}$ = .32 vs. .39, ${\mathrm{UCLA}}_{8Items}$ = .45 vs. .45, ${\mathrm{UCLA}}_{3Items}$ = .37 vs. .41, and SI Direct = .37 vs. .27), indicating that the lower correlations found in Study 2 might be attributed to the lower internal consistency of the abbreviated measures.

As in Study 1, scores of the ${\mathrm{UCLA-LS}}_{8Items}$ showed the highest associations with aspects related to sociability (i.e., extraversion, sociability, shyness, affiliation) and, thus, were similar in this regard to the ${\mathrm{RTLS}}_{Social}$ . By contrast, scores of other scales such as the ${\mathrm{UCLA}}_{3Items}$ or the SI Direct were less strongly or even not significantly associated with aspects of sociability.

#### Satisfaction

On average, there were no strong differences between scores of the loneliness measures with .27 ≤ $\left|\bar{r}\right|$ ≤ .35. The strongest differences between the measures emerged for satisfaction with friends and social contacts. In this domain, scores of the ${\mathrm{RTLS}}_{Total}$ , ${\mathrm{RTLS}}_{Social}$ , and ${\mathrm{UCLA-LS}}_{8Items}$ showed quite strong associations, whereas the association with scores of the remaining measures were lower.

#### Profile Similarity

The profile similarities for Study 2 are displayed in Table 8. The profiles of the different loneliness scores varied between .77 ≤ ICCDE ≤ .99. The highest similarity was found between the SI Direct and the ${\mathrm{UCLA-LS}}_{3Items}$ and the lowest between ${\mathrm{RTLS}}_{Emotional}$ and ${\mathrm{RTLS}}_{Social}$ . Similarly, the profile correlations between ${\mathrm{RTLS}}_{Social}$ and SI Direct (.84) and ${\mathrm{UCLA-LS}}_{3Items}$ (.79) were lower than the profile similarities between other scores.

Comparing the profiles of scores of the measures that were used in both studies (i.e., RTLS and facets, ${\mathrm{UCLA-LS}}_{20Items/8Items}$ , ${\mathrm{UCLA-LS}}_{3Items}$ , SI Direct) regarding the correlates that were used in both studies yielded a high profile similarity. Specifically, the profile similarity between the nomological nets in Study 1 and Study 2 was high for scores of the ${\mathrm{RTLS}}_{Total}$ ( $ICC_{DE}=.92$ ), ${\mathrm{RTLS}}_{Emotional}$ ( $ICC_{DE}=.89$ ), ${\mathrm{RTLS}}_{Social}$ ( $ICC_{DE}=.87$ ), ${\mathrm{UCLA-LS}}_{20Items/8Items}$ ( $ICC_{DE}=.88$ ), ${\mathrm{UCLA-LS}}_{3Items}$ ( $ICC_{DE}=.91$ ), and SI Direct( $ICC_{DE}=.91$ ). Thus, we conclude that the nomological nets of scores of these popular measures are largely invariant across samples and studies (for similar findings regarding the Big Five, see Buecker et al., 2021).

#### Summary and Conclusion

The results of Study 2 compared well with the results obtained in Study 1. We found a similar pattern of convergent validity among scores of different loneliness measures in the self-ratings and, with the exception of SI Direct, also in the partner-ratings. Moreover, we found a very high similarity between the profiles of different loneliness item scores and external correlates across the two studies. Although Study 2 cannot be considered a direct replication of Study 1, the similarity between the results indicates that the findings are robust. Thus, Study 2 further corroborates that scores of all included measures are valid and reliable measures of loneliness in adults.

## Study 3: Reliability of Scores of Single-Item Measures

In Studies 1 and 2, we found that the nomological nets of scores of single-item measures of loneliness overlap less with phenomena related to extraversion and sociability. This finding might point either to purer nomological nets or to a lack of validity of the scores of single items—because of low reliability, their correlation with other constructs might be reduced.

Two previous studies have used STARTS models (Kenny & Zautra, 2001) on single-item scores of loneliness (Mund, Lüdtke, & Neyer, 2020; Zhong et al., 2016). In this way, Zhong et al. (2016) estimated a reliability of .595 for scores of an SI Direct, whereas the results reported by Mund, Lüdtke, and Neyer (2020) imply a reliability of scores of different single-item measures in the range between .455 and .524 . An alternative to STARTS models is a procedure proposed by Heise (1969). In this approach, the reliability of single-item scores is estimated based on their autocorrelations over three evenly spaced measurement occasions. Specifically, Heise (1969) assumed that the stability of a construct depends on the retest-reliability of the scale scores used to measure the construct and the decline in true stability of the construct over time. Using the observed autocorrelations of scale scores across three measurement occasions, it is possible to derive a reliability estimate ( $r_{xx}$ ) that is free of transient or other effects of temporal change (Heise, 1969; McCrae et al., 2011; Robins et al., 2001). In Study 3, we adopted this approach and examined the retest-reliability of the scores of three single-item measures of loneliness.

### Method

#### Sample

In 2019, we recruited a sample of 411 individuals via the service provider Prolific (www.prolific.com; for an overview, see Palan & Schitter, 2018). As the study only involved self-ratings, no ethical approval was necessary according to German regulations; however, all participants provided informed consent before entering the study. On average, participants were 30.06 years old ( SD=9.23 ), ranging from 18 to 63 years ( Mdn=28 ). Almost half of the sample (47.7%) were women. Regarding employment status, 46% and 25% of the sample reported to work full-time or part-time, respectively; 7.2% reported not to be in paid work (e.g., homemaker, disabled, retired). The remaining ≈12% of individuals reported to be currently job seeking, about to start a new job, or “other” status. Most participants were of German (76%) or Austrian (14%) nationality. The Prolific score (possible range from 0 to 100) of the participants, resembling the quality and validity of their responses based on previous studies they participated in, was high (*M* = 99.57, SD=1.22 , Mdn=100 , range from 87 to 100).

Two weeks after the first assessment (T1), all participants were reinvited to participate a second time (T2). Of the initial 411 individuals, 278 (67%) participated again. Another 2 weeks later (i.e., 4 weeks after the initial assessment), all participants were invited to the third survey (T3) and 276 individuals (67% compared to T1) provided the necessary data. For estimating the reliability of scores of the single items, we conducted an analysis based on all available data and, separately, for the panel sample of 276 individuals who participated in all three assessment points. The data used for the key analyses are available from https://osf.io/7gsfw/.

### Measures

#### Loneliness

As measures of loneliness, we included the three single items also included in Study 1. That is, we included the SI Direct, SI Indirect, and the SI ${\mathrm{SI}\;\mathrm{Direct}}_{frequency}$ . Descriptive information for all measures is displayed in Supplemental Table S9.

#### Correlates

The three single-item measures of loneliness were interspersed with other measures. We included the three-item measure of *self-esteem* developed for large panel studies (Huinink et al., 2011) that was also used in the present Study 1. Furthermore, participants rated the frequency of being *tense*, *joyful*, *sad*, and *happy*. We will consider scores of self-esteem in the following analyses, but the emotion ratings were merely used as fillers and will not be considered further.

#### Analytic Strategy

The procedure described by Heise (1969, Formula 9) requires three autocorrelations among the items measuring loneliness: the correlation between scores of T1 and T2 ( $r_{12}$ ), T1 and T3 ( $r_{13}$ ), and T2 and T3 ( $r_{23}$ ). Based on these three correlations, reliability is estimated as $r_{xx}=(r_{12}\times r_{23})/r_{13}$ . We applied this formula separately to scores of all three single-item measures. Moreover, we applied the formula to the point estimate of the observed correlations and additionally to the lower and upper bound of the 95% confidence interval of the observed correlations.

### Results and Discussion

Before estimating the reliability of scores of the single-item measures, we investigated their correlation with scores of the brief self-esteem measure. The correlation between self-esteem scores and scores of SI Direct (*r*T1 = –.55, *r*T2 = –.51, *r*T3 = –.51), SI Indirect ( $r_{T1}=-.53,r_{T2}=-.53,r_{T3}=-.45$ ), and ${\mathrm{SI}\;\mathrm{Direct}}_{frequency}$ ( $r_{T1}=-.51,r_{T2}=-.52,r_{T3}=-.45$ ) converged well with the coefficients observed in the present Studies 1 and 2.

Table 9 displays the reliability of the scores of single-item measures of loneliness for the full and panel sample. The highest reliability was estimated for scores of the SI Indirect, followed by scores of the ${\mathrm{SI}\;\mathrm{Direct}}_{frequency}$ . The reliability of scores of the SI Direct was somewhat lower, but still above typically used cut-offs of .70. Thus, scores of these three single-item measures have a reliability that can at least be considered adequate. The often-cited notion that scores of single items are unreliable appears questionable against the backdrop of the results of this analysis. Furthermore, the reliability estimated in this study is similar to reliability estimates for scores of single items of self-esteem ( $r_{xx}=.75$ using the Heise approach; Robins et al., 2001) and life satisfaction (average $r_{xx}$ across four independent studies = .72 using bivariate STARTS models; Lucas & Donnellan, 2012). Thus, it seems possible to reliably capture loneliness in adults using scores of single-item measures. This finding is particularly important when financial or time-related constraints do not allow researchers to include multi-item measures of loneliness.

**Table 9.** Study 3: Reliability of Scores of Single-Item Measures.

Scale	Full (*N* = 411)			Panel (*N* = 276)			Correlations at T1^a^		
	$r_{xx}$	LB	UB	$r_{xx}$	LB	UB	1	2	3
1 SI Direct	.71	.65	.76	.74	.66	.77		.79	.82
2 SI Indirect	.82	.77	.85	.85	.81	.88	.80		.72
3 ${\mathrm{SI}\;\mathrm{Direct}}_{frequency}$	.77	.71	.81	.80	.74	.84	.81	.74	

*Note.* $r_{xx}$ = reliability as estimated by Formula 9 presented in Heise1969; LB = lower bound of the 95% confidence interval of the estimated reliability; UB = upper bound of the 95% confidence interval of the estimated reliability; SI = single item.

a Correlations in the Full Sample are displayed below the diagonal, and correlations in the Panel Sample are displayed above the diagonal.

## General Discussion

In the present studies, we examined the psychometric features of scores of several measures of loneliness—the perception of one’s relationships as deficient regarding the quantitative and/or qualitative aspects (Ernst & Cacioppo, 1999; Perlman & Peplau, 1981). We included versions of the RTLS, UCLA-LS, and different single-item measures because those are among the most popular and widely used measures of loneliness in adulthood (Buecker et al., 2020; Maes et al., 2019; Mund, Freuding, et al., 2020; Pinquart & Sörensen, 2001). At the same time, these measures represent broader operationalizations of loneliness: The RTLS represents an indirect multidimensional approach, whereas the UCLA-LS represents an indirect unidimensional approach to loneliness. The included single-item measures are necessarily unidimensional, but represent both direct and indirect approaches. This study largely extends previous research by (a) focusing on several loneliness measures at once, (b) using self-ratings and informant-ratings to assess loneliness, (c) examining and comparing broad nomological nets, and (d) paying attention to the validity and reliability of different versions of single-item measures. The results presented in this article offer many vantage points for discussion, including (a) the similarities and differences between measures of loneliness, (b) the debate around the use of single items, and (c) recommendations for future research.

### Similarities and Differences Between Loneliness Measures

In Study 1 and Study 2, we investigated the convergent validity of scores of different measures of loneliness, self-informant agreement, and the nomological nets of these item scores. The results indicate that scores of all measures included in this study are useful measures of loneliness. All item scores show high convergent validity in the self-reports and informant-reports. Furthermore, the nomological nets of scores of all measures were very similar to each other as indicated by high profile correlations. However, it should be noted that consistent across Studies 1 and 2, scores of the UCLA-LS, in its 20-item as well as in its 8-item version, were more strongly related to affiliative aspects like sociability and extraversion than scores of other measures, even after controlling for unreliability in other item scores. Similarly, scores of the ${\mathrm{RTLS}}_{Social}$ were more strongly related to sociability, extraversion (Studies 1 and 2), and network characteristics such as the frequency of joint activities with friends (Study 1). By contrast, scores of the ${\mathrm{RTLS}}_{Emotional}$ were more strongly related to variables such as neuroticism and self-esteem. Thus, the findings for scores of the RTLS support the idea that, despite the high intercorrelation between scores of its facets, it allows to differentiate between emotional and social aspects of loneliness (de Jong Gierveld & Kamphuis, 1985; de Jong Gierveld & van Tilburg, 2006; Green et al., 2001; van Baarsen et al., 2001). The correlation between scores of the RTLS and UCLA-LS with personality and network characteristics demonstrates that the indirect approach taken by both instruments might come along with a less clear separation of loneliness from related constructs (Buecker et al., 2020). As we will discuss below, this approach might have consequences for specific research questions.

Across all measures and both Study 1 and Study 2, depressiveness and life satisfaction emerged as key correlates of loneliness. These findings are in line with prior research suggesting that loneliness might be a risk factor for depression (Cacioppo et al., 2010; Erzen & Çikrikci, 2018) or co-occur due to a common origin (Abdellaoui et al., 2018; Beutel et al., 2017; Cacioppo, Hughes, et al., 2006). The association with lower life satisfaction further suggests that loneliness might have very broad influences on how individuals see and approach the world. More specifically, although loneliness is conceptualized to emerge from perceived deficiencies in one’s social relationships, its consequences seem to reach far beyond social relationships and to cloud individual’s perspective on life more generally (Ernst & Cacioppo, 1999; Perlman & Peplau, 1981).

Consistent with prior research (Lee & Ko, 2018; Luhmann et al., 2016; Mearns et al., 2009), self-informant agreement within and across item scores was generally high and similar to self-informant agreement in broader personality characteristics (Connelly & Ones, 2010). This finding indicates that loneliness can be perceived by others. From a psychometric perspective, this supports the notion that scores of the measures selected in this article capture reliable interindividual differences that are, to a large extent, validated by informants (McCrae et al., 2004; Podsakoff et al., 2003). From an applied perspective, this finding might also indicate that close others can accurately evaluate a person’s loneliness. Thus, campaigns to tackle loneliness might also benefit from equipping close others with the knowledge of how to help and support lonely individuals in their environment in addition to encouraging lonely people to seek help proactively.

It should be noted that the informant-ratings in the present and previous studies (Lee & Ko, 2018; Luhmann et al., 2016; Mearns et al., 2009) were obtained from close others such as partners, friends, or parents. Loneliness might be less well observable for less close informants, such as teachers or casual acquaintances (Geukens et al., 2021). Unlike extraversion, for example, which can be judged accurately even after a few seconds of interaction (Back & Nestler, 2016; Connelly & Ones, 2010; Vazire, 2010), loneliness is a rather internal state that might not manifest readily in specific observable behaviors (see also Vazire, 2010). Instead, loneliness might express itself in the content of interactions, for example, by a specific emotional tone present in repeated conversations, or specific language patterns (Mehl et al., 2017; for findings on neuroticism, see Tackman et al., 2020) or interaction patterns (Tsai & Reis, 2009).

### Debate Around Single-Item Measures

In Study 1 and Study 2, we demonstrated that scores of different versions of single-item measures correlate highly with each other and with scores of multi-item scales. Furthermore, we demonstrated that the nomological nets of scores of single items are consistent across different formulations and with the nomological nets of the established RTLS and UCLA-LS item scores. In Study 3, we also demonstrated that scores of single items are reliable. Thus, the results of the present studies indicate that scores of single-item measures are not deficient or invalid measures of loneliness per se. Consequently, we argue that single-item measures of loneliness have their place as robust and trustworthy measures in research on loneliness.

The practice of measuring loneliness via single items has been controversial (Marangoni & Ickes, 1989; D. Russell et al., 1980). However, single-item measures are highly prevalent in research on loneliness (Mund, Freuding, et al., 2020; Pinquart & Sörensen, 2001). This controversy seems to resemble similar discussions surrounding single-item measures of self-esteem (Robins et al., 2001) and life satisfaction (Cheung & Lucas, 2014; Lucas & Donnellan, 2012). Specifically results in those research fields have suggested that both self-esteem and life satisfaction are so highly schematized constructs that single-item scores are reliable and valid. The results of this study seem to suggest that loneliness is highly schematized as well and can be measured using single items in contexts where financial or time-related constraints prohibit the use of multi-item scales. However, it should also be noted that the use of single-item and other ultra-short measures of loneliness such as the ${\mathrm{UCLA-LS}}_{3Items}$ comes with the disadvantage of losing bandwidth. That is, short measures are very focused measures that cannot capture a construct in all its complexity. Thus, although feasible in many research contexts, single-item and ultra-short measures of loneliness should not be used in clinical practice or in settings where a very nuanced perspective is required (Kemper et al., 2019).

### Recommendations for Future Research

Broadly speaking, when it comes to measuring the core of loneliness, we proclaim the well-known Dodo Verdict that “everybody has won, so all shall have prizes.” Put differently, we believe that the phenomenon of loneliness in adulthood can be measured robustly, validly, and reliably by scores of any of the measures investigated in this article.

A more nuanced view is necessary within specific research contexts. For example, in studies designed to investigate the behavioral correlates and/or the observability of loneliness, researchers need to take care that any observed effects are not due to other factors. For example, most studies that have examined self-informant agreement in loneliness have used scores of the UCLA-LS (Lee & Ko, 2018; Luhmann et al., 2016; Mearns et al., 2009). Given the results presented in the current studies, the high self-informant agreement found in these studies might be attributable, to some extent, to informants having actually observed the target’s extraversion or sociability, which are well observable even for raters who are unacquainted with the target (Back & Nestler, 2016; Connelly & Ones, 2010). As another example, the similarity in loneliness between romantic partners has been found to be moderate ( r=.27 ) using the ${\mathrm{UCLA-LS}}_{8Items}$ (Mund et al., 2022), but to be near zero ( r=.08 ) in a study using a single item (Mund & Johnson, 2021). By contrast, the effects of loneliness on later relationship satisfaction were similar in both studies.

The general recommendation that can be taken from the present findings is that loneliness measures should be selected with a particular regard to the research question. Some of the existing measures (e.g., RTLS, UCLA-LS) might need to be adjusted statistically to rule out confounding with overlapping constructs (e.g., extraversion). Furthermore, some measures might not work well when informant-ratings are to be collected, as was the case for the SI Direct in the present Study 2; in such cases, indirect measures might be better suited. Ideally, researchers should include multiple measures whenever possible to cross-check the results. The nomological nets presented in this study might help to spot potential sources of disagreement between measures in existing and future research.

### Limitations

The findings presented in this article should be evaluated against the backdrop of some limitations. First, recruitment of the participants was conducted via the internet and, thus, was prone to self-selection. Overall, the samples were composed of mostly young and highly educated adults. Hence, it is unclear whether the results can be generalized to samples from other age groups, other educational or cultural backgrounds, or with chronic health conditions.

Second, we investigated a selection of measurement instruments that are frequently used in contemporary research on loneliness in adulthood (Buecker et al., 2020; Maes et al., 2019; Mund, Freuding, et al., 2020; Pinquart & Sörensen, 2001). However, other loneliness measures are also available. Some of those measures have been developed for samples of children or adolescents (Maes et al., 2015, 2017; Marcoen et al., 1987), and some have been developed with a broad multidimensional and relationship-specific perspective (DiTommaso & Spinner, 1993; Hoza et al., 2000; Maes et al., 2015; Pollet et al., 2018). We hope that the nomological nets presented in this study might serve as a foundation for further investigating the construct validity of scores of those measures.

Third, although we investigated comprehensive nomological nets of scores of different loneliness measures, several important correlates were not considered. These include, first and foremost, health behaviors (Eccles et al., 2020; Shankar et al., 2011), but also other phenomena such as externalizing problems (e.g., hostility; Luhmann et al., 2015; Segel-Karpas & Ayalon, 2020) or social media usage (Nowland et al., 2018).

## Conclusion

Across three studies, we investigated aspects of the validity and reliability of scores of different loneliness measures—including several versions of established scales such as the RTLA and the UCLA-LS, but also different versions of single-item measures. Overall, the results indicate that scores of all measures are highly correlated in self-ratings as well as informant-ratings. Furthermore, we found substantial self-informant agreement on scores of all measures. The nomological nets of the included item scores were shown to be consistent within and across studies. Finally, we have shown that scores of single-item measures of loneliness possess adequate reliability. Taken together, the findings of the present studies provide rich evidence for the validity of scores of popular loneliness measures, thereby providing a nuanced picture of the nomological net of each measure’s item scores. Thus, this study might guide further research in selecting an appropriate loneliness measure and at the same time provide a solid foundation for future research on the validity of scores of other loneliness measures.

## Supplemental Material

sj-docx-1-asm-10.1177_10731911221077227 – Supplemental material for Would the Real Loneliness Please Stand Up? The Validity of Loneliness Scores and the Reliability of Single-Item Scores Click here for additional data file. Supplemental material, sj-docx-1-asm-10.1177_10731911221077227 for Would the Real Loneliness Please Stand Up? The Validity of Loneliness Scores and the Reliability of Single-Item Scores by Marcus Mund, Marlies Maes, Pia M. Drewke, Antonia Gutzeit, Isabel Jaki and Pamela Qualter in Assessment
