# Supplementary material for: Would the Real Loneliness Please Stand Up? The Validity of Loneliness Scores and the Reliability of Single-Item Scores
Source: Assessment. 2022 Mar 4;30(4):1226–48. doi: 10.1177/10731911221077227 (PMC10149889; doi:10.1177/10731911221077227)
Supplement: sj-docx-1-asm-10.1177_10731911221077227 – Supplemental material for Would the Real Loneliness Please Stand Up? The Validity of Loneliness Scores and the Reliability of Single-Item Scores [file sj-docx-1-asm-10.1177_10731911221077227.docx]

Would the Real Loneliness Please Stand Up? The Validity of Loneliness Scores and the Reliability of Single-Item Scores

PSYCHOMETRICS OF LONELINESS | SUPPLEMENT 2

# Study 1

**Table S1**

*Overview of the Measures Used in Study 1*

Coefficient *ω*

Construct Source Sample Item Scale Range # Items Self Informant

*Demographic Characteristics*

Age “How old are you?” 18–100 in 1-year steps

Gender “Please indicate your

PSYCHOMETRICS OF LONELINESS | SUPPLEMENT

3

gender”

Education “Please select your

highest educational degree”

Partnered “Are you involved in a

partner relationship?”

1 (male), 2 (female), 3 (other), 4 (not saying); for the analysis reported in the mansucript, the variable was recoded as 1 (female) and 0 (not female, incl. male and other)

1 (no degree), 2 (Grundschule — Primary School), 3 (Hauptschule

— secondary modern school qualification), 4 (Realschule — secondary school certificate), 5 (Fachhochschulreife — entrance qualification for a university of applied sciences), 6 (Abitur — university entrance qualification), 7 (Hochschulstudium — university degree, e.g., B.Sc., M.Sc.), 8

(Promotion — PhD)

1 (*yes*), 0 (*no*)

Table S1 (continued)

Coefficient *ω*

Construct Source Sample Item Scale Range # Items Self Informant

*Loneliness*

Rasch-Type Loneliness Scale (RTLS*_T_ _otal_*)

Emotional Loneliness (RTLS*Emotional*)

Social Loneliness (RTLS*Social*)

UCLA Loneliness Scale (UCLA-LS_20_*_Items_*)

3-Item UCLA Loneliness

Scale (UCLA-LS_3_*_Items_*)

de Jong Gierveld and Kamphuis [(1985)](#_bookmark6)

de Jong Gierveld and Kamphuis [(1985)](#_bookmark6)

de Jong Gierveld and Kamphuis [(1985)](#_bookmark6)

Döring and Bortz [(1993)](#_bookmark8)

Hawkley et al. [(2015)](#_bookmark13)

“I experience a general sense of emptiness”

“There are plenty of people I can rely on when I have problems”, reverse scored

“Nobody really knows me”

“How often do you have the feeling to be left out”; “How often do you have the feeling to be isolated”; “How often do you have the feeling to lack companionship”

1 (*does not apply*) to 5 (*fully applies*)

1 (*does not apply*) to 5 (*fully applies*)

1 (*does not apply*) to 5 (*fully applies*)

1 (*completely false*) to 5 (*completely true*

1 (*never* ) to 5 (*very often*)

11 .89 .87

6 .80 .83

5 .87 .82

20 .94 .93

3 .78 .80

Direct Single Item (SI

Direct)

Indirect Single Item (SI Indirect)

Döring and Bortz [(1993)](#_bookmark8)

“I feel lonely” 1 (*does not at all apply*) 1

to 5 (*does fully apply*)

“I feel alone” 1 (*does not at all apply*) 1

to 5 (*does fully apply*)

Direct Single Item Frequency (SI

“How often do you feel lonely”

1 (*never* ) to 5 (*always*) 1

Direct*Frequency* )

*Personality*

Neuroticism Hahn et al. [(2012)](#_bookmark12) “I see myself as someone who worries a lot”

PSYCHOMETRICS OF LONELINESS | SUPPLEMENT

4

Extraversion Hahn et al. [(2012)](#_bookmark12) “I see myself as someone who is communicative”

1 (*do not agree at all)* to 7 (*fully agree*)

1 (*do not agree at all)* to 7 (*fully agree*)

3 .76

3 .84

who is original”

| Table S1 (continued) |  | | | | | |
| --- | --- | --- | --- | --- | --- | --- |
|  |  |  |  |  | Coefficient *ω* |  |
| Construct | Source | Sample Item | Scale Range | # Items | Self Informant |  |
| Openness | Hahn et al. [(2012)](#_bookmark12) | “I see myself as someone | 1 (*do not agree at all)* to | 3 | .68 |  |

Agreeableness Hahn et al. [(2012)](#_bookmark12) “I see myself as someone

who has a forgiving nature”

Conscientiousness Hahn et al. [(2012)](#_bookmark12) “I see myself as someone

who does things effectively”

7 (*fully agree*)

1 (*do not agree at all)* to 7 (*fully agree*)

1 (*do not agree at all)* to 7 (*fully agree*)

3 .54

3 .71

Self-Esteem German Family Panel

Depressiveness Spaderna et al. [(2002)](#_bookmark23)

Affiliation Motive Schönbrodt and Gerstenberg [(2012)](#_bookmark21)

“I like myself just the way I am”

“In general, how often do you feel sad”

“How important is it for you to be in the company of friends?”

1 (*do not agree at all)* to 5 (*fully agree*)

1 (*almost never* ) to 4 (*almost always*)

1 (*not important*) to 6 (*very important*)

3 .80

10 .91

4 .73

Need Satisfaction Chen et al. [(2015)](#_bookmark5) “In general, people are

friendly with me”, reverse scored

1 (*does not apply*) to 5 (*applies fully*)

2 .66

Social Desirability Stöber [(2001)](#_bookmark25) “In traffic I am always

polite and considerate of others”

true/false 17 .71

Shyness Asendorpf and Wilpers [(1998)](#_bookmark2)

Sociability Asendorpf and Wilpers [(1998)](#_bookmark2)

“I feel inhibited when I am with other people” “I really like to talk to other people”

1 (*does not apply*) to 5 (*applies fully*)

1 (*does not apply*) to 5 (*applies fully*)

5 .76

5 .79

*Satisfaction*

PSYCHOMETRICS OF LONELINESS | SUPPLEMENT

5

Life Siedler et al. [(2008)](#_bookmark22) “In general, how satisfied are you with your life”

Education Siedler et al. [(2008)](#_bookmark22) “In general, how satisfied are you with your education and work life”

0 (*very dissatisfied*) to 10 1

(*very satisfied*)

0 (*very dissatisfied*) to 10 1

(*very satisfied*)

are you with your leisure”

| Table S1 (continued) |  | | | | |
| --- | --- | --- | --- | --- | --- |
|  |  |  |  | Coefficient *ω* |  |
| Construct | Source | Sample Item | Scale Range | # Items Self Informant |  |
| Leisure | Siedler et al. [(2008)](#_bookmark22) | “In general, how satisfied | 0 (*very dissatisfied*) to 10 | 1 |  |

Friends Siedler et al. [(2008)](#_bookmark22) “In general, how satisfied are you with your friends and social contacts”

Family Siedler et al. [(2008)](#_bookmark22) “In general, how satisfied are you with your family relationships”

Relationship Siedler et al. [(2008)](#_bookmark22) “In general, how satisfied are you with your partner

(*very satisfied*)

0 (*very dissatisfied*) to 10 1

(*very satisfied*)

0 (*very dissatisfied*) to 10 1

(*very satisfied*)

1. (*very dissatisfied*) to 10 1

(*very satisfied*)

relationship”

*Network Characteristics*

# close friends 1

# friends on Facebook 1

# persons to discuss problems with

PSYCHOMETRICS OF LONELINESS | SUPPLEMENT

6

# persons to contact for practical help

# persons to ask for advice

Contact frequency with closest friend

1

1

1

*once a year* (1), *every few* 1

*months* (2), *once a month* (3), *two to three times per month* (4), *once a week* (5), *several times a week* (6), *daily* (7)

Table S1 (continued)

Coefficient *ω*

Construct Source Sample Item Scale Range # Items Self Informant

Frequency of joint activities with friends

PSYCHOMETRICS OF LONELINESS | SUPPLEMENT

7

*Note.* Informant-ratings were only collected for the loneliness measures.

*once a year* (1), *every few* 1

*months* (2), *once a month* (3), *two to three times per month* (4), *once a week* (5), *several times a week* (6), *daily* (7)

## PSYCHOMETRICS OF LONELINESS | SUPPLEMENT 8

**Table S2**

*Descriptive Statistics*

Self-Rating Informant-Rating*a*

Measure M SD Md M SD Md

| *Loneliness* |  | | | | | | |
| --- | --- | --- | --- | --- | --- | --- | --- |
| RTLS*T otal* | | 2.36 | 0.85 | 2.27 | 2.10 | 0.73 | 2.02 |
| RTLS*Emotional* | | 2.47 | 0.93 | 2.33 | 2.24 | 0.86 | 2.17 |
| RTLS*Social* | | 2.22 | 0.94 | 2.00 | 1.94 | 0.69 | 1.80 |
| UCLA20*Items* UCLA3*Items* | | 2.07  2.60 | 0.73  0.85 | 1.90  2.67 | 1.76  2.27 | 0.60  0.76 | 1.57  2.17 |
| SI Direct 2.69 | | | 1.31 | 2.00 | 2.39 | 1.21 | 2.00 |
| SI Indirect 2.77 | | | 1.25 | 2.00 | 2.49 | 1.21 | 2.00 |
| SI Direct*_Frequency_* 2.60 | | | 0.95 | 3.00 | 2.43 | 0.79 | 2.42 |
| *Personality* | |  |  |  |  |  |  |
| Neuroticism | 4.38 | | 1.42 | 4.67 | | | |
| Extraversion | 4.42 | | 1.45 | 4.67 | | | |
| Openness | 4.99 | | 1.22 | 5.00 | | | |
| Agreeableness | 5.31 | | 1.01 | 5.33 | | | |
| Conscientiousness | 5.00 | | 1.17 | 5.00 | | | |
| Shyness | 2.85 | | 0.71 | 2.80 | | | |
| Sociability | 3.06 | | 0.79 | 3.20 | | | |
| Self-Esteem | 3.49 | | 0.98 | 3.67 | | | |
| Affiliation Motive | 4.66 | | 0.87 | 4.75 | | | |
| Need Frustration | 2.00 | | 0.79 | 2.00 | | | |
| Motive*×*Need | −0.16  2.01 | | 0.79  0.60 | 0.00  1.90 | | | |
| Social Desirability |  | 1.45 | 0.18 | 1.47 |  |  |  |
| Life | 6.60 | | 2.06 | 7.00 | | | |
| Education | 6.47 | | 2.46 | 7.00 | | | |
| Leisure | 6.57 | | 2.23 | 7.00 | | | |
| Friends | 6.71 | | 2.36 | 7.00 | | | |
| Family | 7.28 | | 2.48 | 8.00 | | | |
| Partner Relationship | 8.00 | | 1.99 | 8.00 | | | |
| *Network Characteristics* |  | |  |  | | | |
| Overall | 5.35 | | 4.10 | 4.00 | | | |
| on Facebook | 270.84 | | 210.93 | 201.50 | | | |
| Help with Problems | 6.82 | | 5.92 | 5.00 | | | |
| Instrumental Support | 10.07 | | 8.29 | 8.00 | | | |
| Advice | 9.07 | | 11.00 | 6.00 | | | |
| *Frequency of. . .* |  |  |  |  |  |  |  |
| Contact | 5.53 | | 1.33 | 6.00 | | | |
| Joint Activities | 4.15 | | 1.52 | 4.00 | | | |

Depressiveness

*Satisfaction with. . .*

*Number of Friends (Providing). . .*

*Note. a*Informant-ratings were only collected for the loneliness measures. RTLS: Rasch-Type Loneliness Scale. UCLA-LS: University of Los Angeles California Loneli- ness Scale. SI: Single Item.

PSYCHOMETRICS OF LONELINESS | SUPPLEMENT

9

*Frequency of. . .*

| **Table S3**  *Zero-Order Correlations* |  | | | | | | | | | | | | | | | | | | | | | | | |
| --- | --- | --- | --- | --- | --- | --- | --- | --- | --- | --- | --- | --- | --- | --- | --- | --- | --- | --- | --- | --- | --- | --- | --- | --- |
| Measure | 1 | 2 | 3 | 4 | 5 | 6 | 7 | 8 9 10 11 12 13 14 15 16 17 18 19 20 21 22 23 24 25 26 | | | | | | | | | | | | | | | | |
| *Personality* |  |  |  |  |  |  |  |  | | | | | | | | | | | | | | | | |
| 1 Neuroticism |  |  |  |  |  |  |  |  |  |  |  |  | | | | | | | | | | | | |
| 2 Extraversion | −.21 |  |  |  |  |  |  |  |  |  |  |  | | | | | | | | | | | | |
| 3 Openness | −.04 | .32 |  |  |  |  |  |  |  |  |  |  | | | | | | | | | | | | |
| 4 Agreeableness | −.03 | .07 | .15 |  |  |  |  |  |  |  |  |  | | | | | | | | | | | | |
| 5 Conscientiousness | −.11 | .12 | .12 | .19 |  |  |  |  |  |  |  |  | | | | | | | | | | | | |
| 6 Shyness | .35 | −.68 | −.26 | −.07 | −.15 |  |  |  |  |  |  |  | | | | | | | | | | | | |
| 7 Sociability | −.13 | .55 | .18 | .31 | .10 | −.34 |  |  |  |  |  |  | | | | | | | | | | | | |
| 8 Self-Esteem | −.48 | .30 | .17 | .16 | .27 | −.37 | .21 |  |  |  |  |  | | | | | | | | | | | | |
| 9 Affiliation Motive | .03 | .34 | .15 | .28 | .16 | −.12 | .56 | .11 |  |  |  |  | | | | | | | | | | | | |
| 10 Need Frustration | .28 | −.33 | −.11 | −.29 | −.11 | .33 | −.32 | −.48 | −.23 |  |  |  | | | | | | | | | | | | |
| 11 Motive*×*Need | −.08 | .10 | .05 | .11 | .06 | −.07 | .12 | .12 | .24 | −.12 |  |  | | | | | | | | | | | | |
| 12 Depressiveness | .57 | −.32 | −.13 | −.23 | −.28 | .36 | −.28 | −.72 | −.19 | .48 | −.15 |  | | | | | | | | | | | | |
| 13 Social Desirability | −.10 | −.09 | .02 | .36 | .24 | .01 | .10 | .07 | .13 | −.03 | .06 | −.12 | | | | | | | | | | | | |
| *Satisfaction with. . .* |  |  |  |  |  |  |  |  |  |  |  |  | | | | | | | | | | | | |
| 14 Life | −.33 | .25 | .16 | .15 | .23 | −.28 | .23 | .64 | .22 | −.39 | .17 | −.69 | .02 | | | | | | | | | | | |
| 15 Education | −.24 | .16 | .08 | .10 | .26 | −.21 | .16 | .43 | .10 | −.26 | .10 | −.52 | .05 .60 | | | | | | | | | | | |
| 16 Leisure | −.25 | .16 | .06 | .11 | .16 | −.15 | .10 | .42 | .16 | −.29 | .10 | −.48 | .05 .56 .37 | | | | | | | | | | | |
| 17 Friends | −.21 | .32 | .13 | .20 | .12 | −.29 | .26 | .41 | .31 | −.46 | .14 | −.45 | .00 .56 .31 .56 | | | | | | | | | | | |
| 18 Family | −.11 | .15 | .05 | .20 | .18 | −.11 | .21 | .35 | .29 | −.29 | .15 | −.43 | .08 .51 .31 .39 .49 | | | | | | | | | | | |
| 19 Partner Relationship | −.03 | .01 | .01 | .09 | .15 | .05 | .06 | .22 | .23 | −.11 | .17 | −.30 | .08 .48 .19 .25 .33 .31 | | | | | | | | | | | |
| *Network Characteristics* |  |  |  |  |  |  |  |  |  |  |  |  |  | | | | | | | | | | | |
| *Number of Friends (Providing). . .* | | | | | | | | | | | | | | | | | | | | | | | | |
| 20 Overall | −.07 | .24 | .13 | .15 | −.02 | −.16 | .30 | .07 | .26 | −.22 | .06 | −.14 | .01 | .12 | .02 | .09 | .28 | .15 | .02 | | | | | |
| 21 on Facebook | −.01 | .28 | .09 | .04 | .02 | −.17 | .22 | .03 | .18 | −.08 | .07 | −.02 | −.03 | .07 | .01 | −.08 | .07 | .09 | .02 .21 | | | | | |
| 22 Help with Problems −.09 | | .19 | .14 | .15 | .05 | −.17 | .25 | .13 | .21 | −.25 | .03 | −.19 | −.05 | .26 | .08 | .17 | .31 | .24 | −.08 | .37 | .09 | | | |
| 23 Instrumental Support −.17 | | .26 | .15 | .17 | .03 | −.21 | .29 | .18 | .23 | −.26 | .05 | −.27 | .04 | .22 | .13 | .17 | .25 | .25 | .07 | .38 | .12 .51 | | | |
| 24 Advice −.04 | | .14 | .05 | .09 | .01 | −.07 | .17 | .07 | .09 | −.12 | .03 | −.16 | −.03 | .17 | .08 | .08 | .17 | .17 | .01 | .30 | .13 | .47 | .48 |  |
| 25 Contact .01 | | .20 | .11 | .12 | .01 | −.08 | .21 | .03 | .23 | −.13 | .06 | −.10 | −.04 | .02 | .01 | .09 | .22 | .08 | .02 | .21 | .07 | .13 | .15 | .09 |
| 26 Joint Activities −.11 | | .28 | .17 | .13 | .04 | −.15 | .37 | .16 | .39 | −.27 | .12 | −.22 | −.01 | .19 | .12 | .23 | .38 | .22 | .10 | .34 | .21 | .27 | .35 | .15 .52 |

PSYCHOMETRICS OF LONELINESS | SUPPLEMENT 10

# Study 2

**Table S4**

*Overview of the Measures Used in Study 2*

Coefficient *ω*

Construct Source Sample Item Scale Range # Items Self Informant

*Demographic Characteristics*

Age “Please indicate your

PSYCHOMETRICS OF LONELINESS | SUPPLEMENT

11

birth year”

Gender “Please indicate your

gender”

Due to the high frequency of measurement occasions every three months, we decided to ask for birth year so that particiants were not required to indicate their age every three months. Instead,

we calculated age by subtracting the year of data collection (e.g., 2020 for Wave 1) from the indicated birth year

1 (male), 2 (female), 3 (diverse); for the analysis reported in the mansucript, the variable was recoded as 1 (female) and 0 (not female, incl. male and diverse)

Relationship Duration “How long have you been involved in the relationship with your current partner?”

| Table S4 (continued) |  | | | | | |
| --- | --- | --- | --- | --- | --- | --- |
|  |  |  |  |  | Coefficient *ω* |  |
| Construct | Source | Sample Item | Scale Range | # Items | Self Informant |  |
| Education |  | “Please select your | 1 (no degree), 2 |  | | |
|  |  | highest educational | (Grundschule — Primary |  |  |  |
|  |  | degree” | School), 3 (Hauptschule |  |  |  |
|  | | | — secondary modern |  |  |  |
|  |  |  | school qualification), 4 |  |  |  |
|  |  |  | (Realschule — secondary |  |  |  |
|  |  |  | school certificate), 5 |  |  |  |
|  |  |  | (Fachhochschulreife — |  |  |  |
|  |  |  | entrance qualification for |  |  |  |
|  |  |  | a university of applied |  |  |  |
|  |  |  | sciences), 6 (Abitur — |  |  |  |
|  |  |  | university entrance |  |  |  |
|  |  |  | qualification), 7 |  |  |  |
|  |  |  | (Hochschulstudium — |  |  |  |
|  |  |  | university degree, e.g., |  |  |  |
|  |  |  | B.Sc., M.Sc.), 8 |  |  |  |

*Loneliness*

(Promotion — PhD) participants could enter a number in a numeric field and select whether they refer to weeks, months, or years; for the analysis reported in the mansucript, we transformed relationship duration to years

Rasch-Type Loneliness Scale (RTLS*_T_ _otal_*)

PSYCHOMETRICS OF LONELINESS | SUPPLEMENT

12

de Jong Gierveld and van Tilburg [(2006)](#_bookmark7)

1 (*does not apply*) to 5 (*fully applies*)

6 .78 .79

[(2006)](#_bookmark7)

| Table S4 (continued) |  | | | | | | |
| --- | --- | --- | --- | --- | --- | --- | --- |
|  |  |  |  |  | Coefficient *ω* | |  |
| Construct | Source | Sample Item | Scale Range | # Items | Self Informant | |  |
| Emotional Loneliness | de Jong Gierveld | “I experience a general | 1 (*does not apply*) to 5 | 3 | .68 .66 | |  |
| (RTLS*Emotional*) | and van Tilburg | sense of emptiness” | (*fully applies*) |  |  | |  |
| Social Loneliness | de Jong Gierveld | “There are plenty of | 1 (*does not apply*) to 5 | 3 | .83 | .85 | |
| (RTLS*Social*) | and van Tilburg | people I can rely on when | (*fully applies*) |  |  |  | |
|  | [(2006)](#_bookmark7) | I have problems”, reverse |  |  |  |  | |

UCLA Loneliness Scale (UCLA-LS_8_*_Items_*)

3-Item UCLA Loneliness

Scale (UCLA-LS_3_*_Items_*)

Döring and Bortz [(1993)](#_bookmark8)

Hawkley et al. [(2015)](#_bookmark13)

scored

“People have a hard time getting close to me” “How often do you have the feeling to be left out”; “How often do you have the feeling to be isolated”; “How often do you have the feeling to lack companionship”

1. (*completely false*) to 5 (*completely true*)

1 (*never* ) to 5 (*very often*)

8 .80 .81

3 .77 .81

Direct Single Item (SI Direct)*a*

*Personality*

Neuroticism Rammstedt et al. [(2020)](#_bookmark18)

Extraversion Rammstedt et al. [(2020)](#_bookmark18)

“I feel lonely” 1 (*does not at all apply*) to 5 (*does fully apply*)

“I worry a lot” 1 (*do not agree at all)* to 5 (*fully agree*)

“I tend to take the lead” 1 (*do not agree at all)* to

5 (*fully agree*)

1

3 .74

3 .52

Openness Rammstedt et al. [(2020)](#_bookmark18)

PSYCHOMETRICS OF LONELINESS | SUPPLEMENT

13

Agreeableness Rammstedt et al. [(2020)](#_bookmark18)

Conscientiousness Rammstedt et al. [(2020)](#_bookmark18)

Self-Esteem von Collani and Herzberg [(2003)](#_bookmark27)

“I can get excited about art, music and literature” “I am compassionate, have a soft heart. ”

“I see myself as someone who does things effectively”

“I like myself just the way I am”

1 (*do not agree at all)* to 5 (*fully agree*)

1 (*do not agree at all)* to 5 (*fully agree*)

1 (*do not agree at all)* to 5 (*fully agree*)

1 (*do not agree at all)* to 5 (*fully agree*)

3 .41

3 .50

3 .641

10 .92

[(2002)](#_bookmark23)

| Table S4 (continued) |  | | | | | |
| --- | --- | --- | --- | --- | --- | --- |
|  |  |  |  |  | Coefficient *ω* |  |
| Construct | Source | Sample Item | Scale Range | # Items | Self Informant |  |
| Depressiveness | Spaderna et al. | “In general, how often do | 1 (*almost never* ) to 4 | 5 | .87 |  |

Affiliation Motive Schönbrodt and Gerstenberg [(2012)](#_bookmark21)

Shyness Asendorpf and Wilpers [(1998)](#_bookmark2)

Sociability*b* Asendorpf and Wilpers [(1998)](#_bookmark2)

you feel sad”

“How important is it for you to be in the company of friends?”

“I feel inhibited when I am with other people” “I really like to talk to other people”

(*almost always*)

1 (*not important*) to 6 (*very important*)

1 (*does not apply*) to 5 (*applies fully*)

1 (*does not apply*) to 5 (*applies fully*)

2 .68

5 .74

5 .68

*Satisfaction*

Life Siedler et al. [(2008)](#_bookmark22) “In general, how satisfied are you with your life”

Education Siedler et al. [(2008)](#_bookmark22) “In general, how satisfied are you with your education and work life”

Leisure Siedler et al. [(2008)](#_bookmark22) “In general, how satisfied are you with your leisure”

Friends Siedler et al. [(2008)](#_bookmark22) “In general, how satisfied are you with your friends and social contacts”

Family Siedler et al. [(2008)](#_bookmark22) “In general, how satisfied are you with your family relationships”

0 (*very dissatisfied*) to 10 1

(*very satisfied*)

0 (*very dissatisfied*) to 10 1

(*very satisfied*)

0 (*very dissatisfied*) to 10 1

(*very satisfied*)

0 (*very dissatisfied*) to 10 1

(*very satisfied*)

0 (*very dissatisfied*) to 10 1

(*very satisfied*)

Relationship Sander and Böcker

[(1993)](#_bookmark19)

“In general, how satisfied

are you with your partner relationship”

0 (*very dissatisfied*) to 5

(*very satisfied*)

7 .81

*Note.* Informant-rating were only collected for the loneliness measures. *a*Self-ratings of the single-item were not collected in Wave 4 (October 2020). *b*Sociability was only assessed at Waves 1 through 3 (i.e., Janury 2020 until July 2020).

PSYCHOMETRICS OF LONELINESS | SUPPLEMENT

14

PSYCHOMETRICS OF LONELINESS | SUPPLEMENT 15

# Table S5

*Descriptive Statistics*

Self-Rating Informant-Rating*a*

| Measure | M | SD | Md |  | M | SD | Md |  |
| --- | --- | --- | --- | --- | --- | --- | --- | --- |
| *Loneliness*  RTLS*T otal* | 2.07 | 0.72 | 2.00 |  | 2.30 | 0.76 | 2.17 |  |
| RTLS*Emotional* | 2.21 | 0.87 | 2.00 | 2.39 | | 0.87 | 2.33 | |
| RTLS*_Social_* 1.93 | | 0.82 | 2.00 | 2.21 | | 0.90 | 2.00 | |
| UCLA_8_*_Items_* 1.98 | | 0.60 | 1.88 | 2.13 | | 0.67 | 2.00 | |
| UCLA_3_*_Items_* 2.22 | | 0.74 | 2.00 | 2.32 | | 0.79 | 2.33 | |
| SI Direct 1.69 | | 0.89 | 1.00 | 1.99 | | 1.02 | 2.00 | |
| *Personality*  Neuroticism | 2.88 | 0.90 | 2.67 | | | | | |
| Extraversion | 3.10 | 0.70 | 3.00 | | | | | |
| Openness | 3.78 | 0.68 | 3.67 | | | | | |
| Agreeableness | 3.67 | 0.72 | 3.67 | | | | | |
| Conscientiousness | 3.36 | 0.77 | 3.33 | | | | | |
| Shyness | 2.82 | 0.63 | 2.80 | | | | | |
| Sociability | 3.09 | 0.68 | 3.20 | | | | | |
| Self-Esteem | 3.31 | 0.81 | 3.50 | | | | | |
| Affiliation Motive | 4.05 | 0.95 | 4.00 | | | | | |
| Depressiveness | 1.81 | 0.57 | 1.80 | | | | | |
| *Satisfaction with. . .*  Life | 7.16 | 2.10 | 8.00 | | | | | |
| Education | 6.49 | 2.73 | 7.00 | | | | | |
| Leisure | 6.58 | 2.49 | 7.00 | | | | | |
| Friends | 7.14 | 2.39 | 8.00 | | | | | |
| Family | 7.34 | 2.61 | 8.00 | | | | | |
| Partner Relationship | 4.43 | 0.47 | 4.57 | | | | | |

*Note. a*Informant-ratings were only collected for the loneliness measures. RTLS: Rasch-Type Loneliness Scale. UCLA-LS: University of Los Angeles California Loneliness Scale. SI: Single Item.

PSYCHOMETRICS OF LONELINESS | SUPPLEMENT

16

**Table S6**

*Zero-Order Correlations*

Measure 1 2 3 4 5 6 7 8 9 10 11 12 13 14 15 16

*Personality*

1. Neuroticism
2. Extraversion −.14
3. Openness .01 .14

| 4 Agreeableness | −.11 | −.02 | .10 |  | | | | | | | | |
| --- | --- | --- | --- | --- | --- | --- | --- | --- | --- | --- | --- | --- |
| 5 Conscientiousness | −.15 | .21 | −.03 | .10 | | | | | | | | |
| 6 Shyness | .25 | −.53 | −.13 | −.06 −.10 | | | | | | | | |
| 7 Sociability | −.08 | .34 | .04 | .25 .03 −.29 | | | | | | | | |
| 8 Self-Esteem | −.63 | .24 | .06 | .14 | .24 | −.29 | .11 | | | | | |
| 9 Affiliation Motive | −.08 | .37 | .04 | .24 | .08 | −.22 | .66 .13 | | | | | |
| 10 Depressiveness | .70 | −.24 | .08 | −.15 | −.18 | .27 | −.17 −.66 −.16 | | | | | |
| 1. Life 2. Education | −.36  −.33 | .19  .16 | .02  .01 | .17  .11 | .15  .19 | −.19  −.14 | .09  .08 | .48  .42 | .11  .13 | −.45  −.40 .61 | | |
| 13 Leisure | −.30 | .17 | .05 | .05 | .09 | −.16 | .07 | .31 | .09 | −.35 | .54 | .39 |
| 14 Friends | −.25 | .20 | .02 | .10 | .10 | −.22 | .08 | .31 | .17 | −.34 | .56 | .34 .62 |
| 15 Family | −.22 | .11 | −.05 | .13 | .06 | −.11 | .09 | .24 | .08 | −.29 | .46 | .29 .37 .44 |
| 16 Partner Relationship | −.16 | .04 | .05 | .13 | .10 | −.06 | −.02 | .21 | .00 | −.26 | .26 | .09 .15 .25 .16 |

*Satisfaction with. . .*

PSYCHOMETRICS OF LONELINESS | SUPPLEMENT 17

# Differences Between Samples in Study 1 and Study 2

## PSYCHOMETRICS OF LONELINESS | SUPPLEMENT 18

**Table S7**

*Comparison of Loneliness Scores Within Study 1 and Study 2*

Study 1 Cohen’s *d*

| Measure | Singles | Partnered | Study 2 | Single vs.Partnered (Study 1) | Partnered (Study 1) vs. Study 2 |  |
| --- | --- | --- | --- | --- | --- | --- |
| RTLS*T otal* | 2.52 (0.84) | 2.21 (0.82) | 2.07 (0.72) | 0.37∗∗∗ | 0.19∗∗ |  |
| RTLS*Emotional* | 2.67 (0.91) | 2.29 (0.90) | 2.21 (0.82) | 0.42∗∗∗ | 0.10 |  |
| RTLS*Social* | 2.33 (0.96) | 2.11 (0.90) | 1.93 (0.82) | 0.24∗∗ | 0.21∗∗∗ |  |
| UCLA20*Items/*8*Items* | 2.21 (0.75) | 1.94 (0.69) | 1.98 (0.60) | 0.37∗∗∗ | −0.06 |  |
| UCLA3*Items* | 2.70 (0.85) | 2.52 (0.84) | 2.22 (0.74) | 0.21∗∗ | 0.39∗∗∗ |  |
| SI Direct | 3.02 (1.30) | 2.39 (1.24) | 1.69 (0.89) | 0.50∗∗∗ | 0.72∗∗∗ |  |
| Average |  |  |  | 0.35 | 0.28 |  |

*Note.* Numbers display mean scores, standard deviations are reported in parentheses. Statistical significance was determined via a two sample Welch test (comparison within Study 1) and using a one-sample *t*-test (Study 1 vs. Study 2). ∗*p <* 0*.*05*,*^∗∗^ *p < .*01*,*^∗∗∗^ *p < .*001.

Although the samples in Study 1 and Study 2 were similar in some respects, several differences emerged regarding their levels of loneliness. In this Supplement, we discuss some of these differences. It should be noted that all participants in Study 2 were partnered, whereas only 50.81% (n = 347) of the participants were partnered in Study 1. A large body of research has demonstrated that partnered individuals report lower average loneliness than individuals without a partner (Dykstra & Fokkema, [2007;](#_bookmark9) Flora & Segrin, [2000;](#_bookmark10) Green et al., [2001;](#_bookmark11) Luhmann & Hawkley, [2016;](#_bookmark16) Stack, [1998;](#_bookmark24) Tornstam, [1992).](#_bookmark26) When comparing the partnered subsample to the subsample of singles within Study 1, the mean scores for the former are substantially lower (see Table [S7).](#_bookmark0)

Overall, the differences between samples decrease when comparing only those being in a relationship. The remaining differences between partnered participants in Study 1 and those of Study 2 might be due to almost 75% of participants in Study 1 being students, whereas the sample in Study 2 was somewhat more diverse with 48% students. Several studies have suggested that loneliness among university students is particularly high (Bauer & Rokach, [2004;](#_bookmark4) Hysing et al., [2020)](#_bookmark15) and also show differences from the general population in other aspects of personality and well- being (Hanel & Vione, [2016;](#_bookmark14) Sanz-Garcia et al., [2021).](#_bookmark20) Indeed, when comparing the partnered students from Study 1 to the students from Study 2, which are all partnered, the differences between samples decrease further, as shown in Table [S8.](#_bookmark1)

The remaining differences between samples might be partially attributable to the dyadic nature of Study 2. Some studies have already shown that dyadic data show somewhat different features than data collected from partnered individuals who participate without their partner. Specifically, it has been shown that partnered participants whose partner also engages in the study report higher levels of relational and individual well-being than partnered individuals whose partner does not engage in the study (Barton et al., [2020;](#_bookmark3) Park et al., [2021).](#_bookmark17)

PSYCHOMETRICS OF LONELINESS | SUPPLEMENT 19

# Table S8

*Comparison Between Partnered Students in Study 1 and Students in Study 2*

| Measure | Study 1 | Study 2 | Cohen’s *d* |
| --- | --- | --- | --- |
| RTLS*T otal* | 2.12 (0.78) | 2.08 (0.74) | 0.05 |

RTLS*_Emotional_* 2.20 (0.85) 2.27 (0.90) −0.08

RTLS*_Social_* 2.01 (0.88) 1.89 (0.82) 0.14∗

UCLA_20_*_Items/_*_8_*_Items_* 1.85 (0.64) 2.00 (0.61) −0.24∗∗∗

UCLA_3_*_Items_* 2.48 (0.83) 2.28 (0.67) 0.28∗∗∗

SI Direct 2.32 (1.19) 1.74 (0.92) 0.58∗∗∗

Average 0.23

*Note.* Numbers display mean scores, standard deviations are reported in parentheses. Statistical significance was determined via a one-sample *t*- test (Study 1 vs. Study 2). ∗*p <* 0*.*05*,*^∗∗^ *p < .*01*,*^∗∗∗^ *p < .*001.

PSYCHOMETRICS OF LONELINESS | SUPPLEMENT 20

# Study 3

PSYCHOMETRICS OF LONELINESS | SUPPLEMENT 21

| **Table S9**  *Descriptive Statistics* |  | | | | | | | | | | |
| --- | --- | --- | --- | --- | --- | --- | --- | --- | --- | --- | --- |
|  | Time 1 |  |  |  | Time 2 |  |  |  | Time 3 |  |  |
| Measure M | SD | Md |  | M | SD | Md |  | M | SD | Md |  |
| SI Direct 2.57 | 1.24 | 2.00 |  | 2.42 | 1.23 | 2.00 |  | 2.41 | 1.20 | 2.00 |  |
| SI Indirect 2.60 | 1.23 | 2.00 |  | 2.50 | 1.21 | 2.00 |  | 2.52 | 1.20 | 2.00 |  |
| SI Direct*_Frequency_* 2.59 | 1.00 | 3.00 |  | 2.59 | 1.01 | 3.00 |  | 2.57 | 0.99 | 3.00 |  |
| *Note.* SI: Single Item |  |  |  |  |  |  |  |  |  |  |  |

PSYCHOMETRICS OF LONELINESS | SUPPLEMENT 22

# References

Asendorpf, J. B., & Wilpers, S. (1998). Personality effects on social relationships. *Journal of Personality and Social Psychology*, *74*, 1531–1544. [https://doi.org/](https://doi.org/10.1037/0022-3514.74.6.1531) [10.1037/0022-3514.74.6.1531](https://doi.org/10.1037/0022-3514.74.6.1531)

Barton, A. W., Lavner, J. A., Stanley, S. M., Johnson, M. D., & Rhoades, G. K. (2020). “Will you complete this survey too?” Differences between individual versus dyadic samples in relationship research. *Journal of Family Psychology*, *34* (2), 196–203. <https://doi.org/10.1037/fam0000583>

Bauer, N., & Rokach, A. (2004). The experience of loneliness in university: A cross- cultural study. *International Journal of Adolescence and Youth*, *11* (4), 283– 302. <https://doi.org/10.1080/02673843.2004.9747936>

Chen, B., Vansteenkiste, M., Beyers, W., Boone, L., Deci, E. L., van der Kaap-Deeder, J., Duriez, B., Lens, W., Matos, L., Mouratidis, A., Ryan, R. M., Sheldon,

K. M., Soenens, B., van Petegem, S., & Verstuyf, J. (2015). Basic psychological need satisfaction, need frustration, and need strength across four cultures. *Motivation and Emotion*, *39*, 216–236. [https://doi.org/10.1007/s11031-014-](https://doi.org/10.1007/s11031-014-9450-1) [9450-1](https://doi.org/10.1007/s11031-014-9450-1)

de Jong Gierveld, J., & Kamphuis, F. H. (1985). The development of a Rasch-type loneliness scale. *Applied Psychological Measurement*, *9*, 289–299. [https://doi.](https://doi.org/10.1177/014662168500900307) [org/10.1177/014662168500900307](https://doi.org/10.1177/014662168500900307)

de Jong Gierveld, J., & van Tilburg, T. (2006). A 6-item scale for overall, emotional, and social loneliness: Confirmatory tests on survey data. *Research on Aging*, *28*, 582–598. <https://doi.org/10.1177/0164027506289723>

Döring, N., & Bortz, J. (1993). Psychometrische Einsamkeitsforschung: Deutsche Neukonstruktion der UCLA Loneliness Scale [Psychometric research on lone- liness: German version of the UCLA loneliness scale]. *Diagnostica*, *39*, 224– 239.

Dykstra, P. A., & Fokkema, T. (2007). Social and emotional loneliness among divorced and married men and women: Comparing the deficit and cognitive perspec- tives. *Basic and Applied Social Psychology*, *29* (1), 1–12. [https://doi.org/10.](https://doi.org/10.1080/01973530701330843) [1080/01973530701330843](https://doi.org/10.1080/01973530701330843)

Flora, J., & Segrin, C. (2000). Relationship development in dating couples: Implica- tions for relational satisfaction and loneliness. *Journal of Social and Personal* *Relationships*, *17* (6), 811–825. <https://doi.org/10.1177/0265407500176006>

Green, L. R., Richardson, D. S., Lago, T., & Schatten-Jones, E. C. (2001). Network correlates of social and emotional loneliness in young and older adults. *Per- sonality and Social Psychology Bulletin*, *27*, 281–288. [https://doi.org/10.1177/](https://doi.org/10.1177/0146167201273002) [0146167201273002](https://doi.org/10.1177/0146167201273002)

Hahn, E., Gottschling, J., & Spinath, F. M. (2012). Short measurement of personality: Validity and reliability of the GSOEP Big Five Inventory (BFI-S). *Journal of Research in Personality*, *46*, 355–359. [https://doi.org/10.1016/j.jrp.2012.03.](https://doi.org/10.1016/j.jrp.2012.03.008)

[008](https://doi.org/10.1016/j.jrp.2012.03.008)

## PSYCHOMETRICS OF LONELINESS | SUPPLEMENT 23

Hanel, P. H. P., & Vione, K. C. (2016). Do student samples provide an accurate estimate of the general public? *PLoS ONE*, *11* (12), e0168354. [https ://doi.](https://doi.org/10.1371/journal.pone.0168354) [org/10.1371/journal.pone.0168354](https://doi.org/10.1371/journal.pone.0168354)

Hawkley, L. C., Duvoisin, R., Ackva, J., Murdoch, J. C., & Luhmann, M. (2015). Loneliness in older adults in the USA and Germany: Measurement invariance and validation. *Working Paper Series, NORC at the University of Chicago*, *Paper 2015–002*.

Hysing, M., Petrie, K. J., Bøe, T., Lønning, K. J., & Sivertsen, B. (2020). Only the lonely: A study of loneliness among university students in Norway. *Clinical* *Psychology in Europe*, *2* (1), 1–16. <https://doi.org/10.32872/cpe.v2i1.2781>

Luhmann, M., & Hawkley, L. C. (2016). Age differences in loneliness from late ado- lescence to oldest old age. *Developmental Psychology*, *52* (6), 943–959. [https:](https://doi.org/10.1037/dev0000117)

[//doi.org/10.1037/dev0000117](https://doi.org/10.1037/dev0000117)

Park, Y., Impett, E. A., & MacDonald, G. (2021). Generalizability of results from dyadic data: Participation of one versus two members of a romantic couple is associated with breakup likelihood. *Personality and Social Psychology Bul-* *letin*, *47* (2), 232–240. <https://doi.org/10.1177/0146167220920167>

Rammstedt, B., Danner, D., Soto, C. J., & John, O. P. (2020). Validation of the short and extra-short forms of the Big Five Inventory-2 (BFI-2) and their German adaptations. *European Journal of Psychological Assessment*, *36* (1), 149–161. <https://doi.org/10.1027/1015-5759/a000481>

Sander, J., & Böcker, S. (1993). Die deutsche Form der Relationship Assessment Scale (RAS) [The German version of the Relationship Assessment Scale (RAS): A short scale for measuring satisfaction in a dyadic relationship]. *Diagnostica*, *39*, 55–62.

Sanz-Garcia, A., Gesteira, C., Sanz, J., & Garcia-Vera, M. P. (2021). Prevalence of psychopathy in the general adult population: A systematic review and meta- analysis. *Frontiers in Psychology*, *12*, 661044. [https://doi.org/10.3389/fpsyg.](https://doi.org/10.3389/fpsyg.2021.661044) [2021.661044](https://doi.org/10.3389/fpsyg.2021.661044)

Schönbrodt, F. D., & Gerstenberg, F. X. R. (2012). An IRT analysis of motive ques- tionnaires: The Unified Motive Scales. *Journal of Research in Personality*, *46* (6), 725–742. <https://doi.org/10.1016/j.jrp.2012.08.010>

Siedler, T., Schupp, J., Spiess, K. C., & Wagner, G. G. (2008). The German Socio- Economic Panel as reference data set. *RatSWD Working Paper*, *48*. [https :](https://doi.org/10.2139/ssrn.1445341)

[//doi.org/10.2139/ssrn.1445341](https://doi.org/10.2139/ssrn.1445341)

Spaderna, H., Schmukle, S. C., & Krohne, H. W. (2002). Bericht über die deutsche Adaption der State-Trait Depression Scales (STDS) [Report about the German adaptation of the State-Trait Depression Scale (STDS)]. *Diagnostica*, *48*, 80– 89. <https://doi.org/10.1026//0012-1924.48.2.80>

Stack, S. (1998). Marriage, family, and loneliness: A cross-national study. *Sociological Perspectives*, *41* (2), 415–432. <https://doi.org/10.2307/1389484>

## PSYCHOMETRICS OF LONELINESS | SUPPLEMENT 24

Stöber, J. (2001). The Social Desirability Scale-17 (SDS-17): Convergent validity, dis- criminant validity, and relationship with age. *European Journal of Psychologi-* *cal Assessment*, *17* (3), 222–232. [https://doi.org/10.1027//1015-5759.17.3.222.](https://doi.org/10.1027//1015-5759.17.3.222)

Tornstam, L. (1992). Loneliness in marriage. *Journal of Social and Personal Rela-* *tionships*, *9*, 197–217. <https://doi.org/10.1177/0265407592092003>

von Collani, G., & Herzberg, P. Y. (2003). Eine revidierte Fassung der deutschsprachi- gen Skala zum Selbstwertgefühl von Rosenberg [A revised version of the Ger- man adaptation of the Rosenberg Self-Esteem Scale]. *Zeitschrift für Differen- tielle und Diagnostische Psychologie*, *24* (1), 3–7.
